# Estimating the causal effects of exposure mixtures: a generalized propensity score method

**DOI:** 10.1186/s12874-025-02673-4

**Published:** 2025-09-29

**Authors:** Qian Gao, Ting Li, Guiming Zhu, Juping Wang, Kexin Qiu, Liangpo Liu, Xiujuan Yang, Tong Wang

**Affiliations:** 1 Department of Health Statistics, School of Public Health, MOE Key Laboratory of Coal Environmental Pathogenicity and Prevention, Shanxi Medical University, No.56 Xinjian South Road, Taiyuan, 030001 Taiyuan China; 2 Department of Public Health Laboratory Sciences, School of Public Health, MOE Key Laboratory of Coal Environmental Pathogenicity and Prevention, Shanxi Medical University, Taiyuan, 030001 China; 3 Department of Statistics, Chinese University of Hong Kong, Shatin, 999077 Hong Kong SAR China

**Keywords:** Multiple exposures, Mixtures, Exposure-response, Causal model, Generalized propensity score

## Abstract

**Background:**

In environmental epidemiology and many other fields, estimating the causal effects of multiple concurrent exposures holds great promise for driving public health interventions and policy changes. Given the predominant reliance on observational data, confounding remains a key consideration, and generalized propensity score (GPS) methods are widely used as causal models to control measured confounders. However, current GPS methods for multiple continuous exposures remain scarce.

**Methods:**

We proposed a novel causal model for exposure mixtures, called nonparametric multivariate covariate balancing generalized propensity score (npmvCBGPS). A simulation study examined whether npmvCBGPS, an existing multivariate GPS (mvGPS) method, and a linear regression model for the outcome can accurately and precisely estimate the effects of exposure mixtures in a variety of common scenarios. An application study illustrated the analysis of the causal role of per- and polyfluoroalkyl substances (PFASs) on BMI.

**Results:**

The npmvCBGPS achieved acceptable covariate balance in all scenarios. The estimates were close to the true value as long as either the exposure or the outcome model was correctly specified, and the results were less impacted by correlations among mixture components. The accuracy and precision of mvGPS and the linear regression model relied on the correctly specified exposure model and outcome model, respectively. The npmvCBGPS outperformed mvGPS in all scenarios. The npmvCBGPS achieved better covariate balance than mvGPS and provided an overall inverse trend between the PFAS mixtures with BMI.

**Conclusions:**

In this study, we proposed npmvCBGPS to accurately estimate the causal effects of multiple exposure mixtures on health outcomes. Our approach is applicable across various domains, with a particular emphasis on environmental epidemiology.

**Supplementary Information:**

The online version contains supplementary material available at 10.1186/s12874-025-02673-4.

## Background

Diseases with a heavy global burden are largely predicted by behavioral, nutritional, occupational, and environmental exposures [1–4], many of which are modifiable and, consequently, may be potential intervention targets [5]. Among these risk factors, even environmental exposures are complex, and these include chemicals from the natural and built environment, such as air and water pollution [6]. In the real world, given that populations are simultaneously exposed to complex multi-pollutant mixtures, there has been a paradigm shift from the “one-exposure-one-disease” framework to evaluating health effects of exposure mixtures [1, 3]. Each of these exposure components may act independently, synergistically, or antagonistically [7]. Focusing on the relationships between health outcomes and multiple simultaneous exposures is advantageous, including the ability to estimate cumulative health effects and provide evidence for more realistic and potentially more effective public health interventions [1, 8].

The estimation of health effects of exposure mixtures comes with numerous challenges; for instance, the typically high correlations between exposures, and the difficulty in capturing interactions and estimating causal relationships [9–11]. Many epidemiological studies have traditionally focused on estimating the health effects of a single exposure [12], such as perfluoroalkyl and polyfluoroalkyl substances [13, 14]. However, these classical single-exposure analyses cannot determine whether the observed association is attributable to the exposure of interest or to another related exposure not considered in the analysis. They also fail to capture the interactive and cumulative effects of exposure mixtures [11]. Recently, several statistical methods have been developed to address some of the challenges in estimating the health effects of exposure mixtures. For example, both weighted quantile sum regression (WQSR) and Bayesian kernel machine regression (BKMR) are robust in addressing collinearity, and BKMR is also able to estimate nonlinear effects [15, 16]. These two methods have been increasingly used since their proposal [12]. However, these two methods are types of regression models and lack clarity regarding the underlying assumptions and definitions necessary for making causal claims about effects [17–19].

Given the predominant reliance on observational data, a major barrier to estimating the causal effects of exposure mixtures is confounding. Classical causal inference methods for confounding adjustment, such as g-computation and generalized propensity score (GPS) methods, have been extended to multiple continuous exposures [7, 20]. Keil et al. proposed quantile g-computation, which combined WQS regression and g-computation [7]. Thilakaratne et al. estimated the associations of nonessential and essential metals with child cognition by using quantile g-computation [kara^21^]. Williams et al. proposed the multivariate generalized propensity score (mvGPS) method [20]. Traini et al. evaluated the causal effects of air pollutant mixtures on overall mortality [17]. Cai et al. built mvGPS models to provide mixture effects of endocrine-disrupting chemicals on metabolic outcomes in adolescents [22]. However, both mvGPS and quantile g-computation are sensitive to model misspecification [17, 23]. In reality, it is usually unknown which model to use to decipher the mechanisms underlying the relationship between confounders and health outcomes or exposures. Oulhote et al. combined super learner (SL) and g-computation (SL-Gcomp) to reduce potential model misspecifications, but SL ignores the causal structure, and the choice of prediction algorithms, and the number of folds of cross-validation in SL may affect the bias and variance of the estimates [24–26]. In this study, we developed a novel GPS approach for handling potential model misspecification, which we call nonparametric multivariate covariate balancing generalized propensity score (npmvCBGPS). The imbalance of confounders across different levels of exposure is a source of confounding bias [23]. The npmvCBGPS method is a weighting method and avoids specifying the GPS/exposure model by directly obtaining weights that optimize “covariate balance”.

This article is organized as follows. First, we conduct a brief review of the inverse probability of weighting (IPW) method. Subsequently, we introduce the proposed npmvCBGPS approach. Following this, we undertake a simulation study to evaluate the validity of the npmvCBGPS. After the analysis, we provide a detailed illustration of the practical application of the npmvCBGPS. Finally, we discuss the results and present conclusions.

## Methods

### Overview of the inverse probability of weighting method

#### Inverse probability of weighting using propensity score

GPS methods are an extension of propensity score (PS) methods, which were originally designed for estimating the causal effect of a single binary exposure [27]. PS methods are widely used in environmental health studies [9]. The inputs of PS methods consist of an observed outcome (*Y*), an exposure (*T*), and a set of covariates (***X***) including confounders and prognostic covariates (those only predicting the outcome). PS methods start by constructing a PS model (or exposure model), and a commonly used model is a logistic model expressed by:

1 $$\:logit\left(P\left(T=1|\varvec{X}\right)\right)={\alpha\:}_{0}+\sum\:_{j=1}^{p}{\alpha\:}_{j}{X}_{j}$$

where $\:{\alpha\:}_{0}$ is the model intercept, and $\:{\alpha\:}_{j}$ is the coefficient of the *j*th covariate. The output of the PS model is the conditional probability that the *i*th individual is assigned to the exposure group (*T* = 1) given $\:{\varvec{X}}_{\varvec{i}}$. This conditional probability is called the propensity score (PS). When the PS is correctly calculated, the PS will be a balancing score meaning that covariates will be balanced between the exposure group (*T* = 1) and the control group (*T* = 0) in subsamples with similar PS [28]. Four different methods using the PS have been widely used to control for measured confounders: propensity score matching, stratification on the propensity score, covariate adjustment using the propensity score, and inverse probability of weighting using the propensity score. Among these PS methods, IPW is increasingly preferred due to its flexibility for different study designs [29].

In IPW analyses, the contribution of the *i*th individual is weighted by the inverse probability weighting (or balance weight) $\:{w}_{i}=\frac{{T}_{i}}{P\left({T}_{i}=1|{\varvec{X}}_{\varvec{i}}\right)}+\frac{1-{T}_{i}}{1-P\left({T}_{i}=1|{\varvec{X}}_{\varvec{i}}\right)}$. The weights ensure that the total contribution is the same between the exposed and control groups for a particular value of the PS [30]. For example, assume there are 10 individuals with a PS of 0.6, including 6 in the exposed group and 4 in the control group. The weight is $\:1/0.6$ for each individual in the exposed group and $\:1/\left(1-0.6\right)=1/0.4$ for each individual in the control group. The sum of weights of 6 individuals in the exposed group, $\:6\text{*}\left(\frac{1}{0.6}\right)$=10 equals the sum of weights of 4 individuals in the control group, $\:4\text{*}\left(\frac{1}{0.4}\right)$=10. Thus, IPW generates a pseudo-population in which covariates are balanced between exposed and control groups without loss of sample. Eventually, we can estimate the average exposure effect by building a marginal structural model expressed as:

2 $$\begin{array}{cc}E\left(Y\left(t\right)\right)={\beta\:}_0+{\beta\:}_1t,&t\in\:\mathcal T\end{array}$$

where $\:\mathcal{T}\subseteq\:\mathcal{R}$ is the support of the exposure and $\:Y\left(t\right)$ is the potential outcome. For a binary exposure, $\:\mathcal{T}=\left\{\text{0,1}\right\}$ and $\:{\beta\:}_{1}=E\left(Y\left(1\right)-Y\left(0\right)\right)$ is the average exposure effect of interest. There are a pair of potential outcomes for each individual: $\:Y\left(1\right)$, which is the outcome they would have had if they had been exposed, and $\:Y\left(0\right)$, which is the outcome they would have had if they had been unexposed. However, in reality, only one outcome can be observed for an individual. Fortunately, under the standard causal identification assumptions (as described in the following paragraph), the observed outcome *Y* and potential outcome $\:Y\left(t\right)$ have the following relationship:

3 $$\begin{array}{cc}E\left(wY\vert T=t\right)=E\left(Y\left(t\right)\right),&t\in\mathcal T\end{array}$$

where $\:w$ is the inverse probability weighting. Therefore, when PS is correctly calculated, we can identify an estimate of $\:{\beta\:}_{1}$ by fitting a weighted univariate linear model of the observed outcome *Y* on the exposure *T* as follows:

4 $$\begin{array}{cc}E\left[Y\vert T=t\right]=\beta_0+\beta_1t,&t\in\mathcal T\end{array}$$

Four assumptions are required to identify causal effects from observational data. Consistency means the observed $\:{Y}_{i}$ equals the potential outcome corresponding to the actual exposure status, i.e., $\:{Y}_{i}=Y\left({T}_{i}=t\right)$, for $\:t\in\:\mathcal{T}$. The stable unit treatment value assumption means no interference among observations. The positivity assumption, $\:0<P\left(T=t|\varvec{X}\right)<1$ for $\:t\in\:\mathcal{T}$ means all subjects have a non-zero probability of being assigned to any exposure level given $\:\varvec{X}$. The no unmeasured confounding assumption $Y\left(t\right)\perp\,T\left|X\right.$ for $\:t\in\:\mathcal{T}$ means all covariates affecting both exposure and outcome have been measured and controlled for. When ***T*** is multidimensional, the no unmeasured confounding assumption needs to hold for each element in ***T***.

#### Inverse probability of weighting using generalized propensity score

When the exposure is continuous, GPS is required for IPW analyses. GPS is defined as the conditional probability density of an individual being exposed to a particular level given covariates [31]. GPS can be estimated by constructing a GPS model (or exposure model), such as a linear model expressed by:

5 $$\begin{array}{cc}T=\alpha_0+\sum_{j=1}^p\alpha_jX_j+\epsilon,&T\in\mathbb{R}\;\text{and}\;\epsilon\sim N\left(0,\sigma^2\right)\end{array}$$

where $\:\epsilon\:$ is a mean-zero random error and follows a normal distribution. In this case, GPS for the *i*th individual can be expressed using the probability density function of the normal distribution: $\:{f}_{T|X}\left({T}_{i}=t|{\varvec{X}}_{\varvec{i}}\right)=\frac{1}{\sqrt{2\pi\:{\sigma\:}^{2}}}exp\left(-\frac{\left(t-{\alpha\:}_{0}-\sum\:_{j=1}^{p}{\alpha\:}_{j}{X}_{ij}\right)}{2{\sigma\:}^{2}}\right)$. The contribution of the *i*th individual is weighted by $\:{w}_{i}=\frac{{f}_{T}\left({T}_{i}=t\right)}{{f}_{T|X}\left({T}_{i}=t|{\varvec{X}}_{\varvec{i}}\right)}$, where the denominator is GPS and the numerator is the marginal probability density of the exposure variable, such as a normal distribution. $\:{w}_{i}$ is a stabilized weight to reduce extreme weights [32]. It is worth noting that valid causal estimates of GPS methods not only rely on standard causal identification assumptions, but also depend on adequate specification of GPS model, including the correct specification of both the function of covariates (e.g., $\:{\alpha\:}_{0}+\sum\:_{j=1}^{p}{\alpha\:}_{j}{X}_{j}$ in Eq. 5) and the distribution of the random error (e.g., a normal distribution for $\:\epsilon\:$ in Eq. 5). Any misspecification in either the function of covariates or the distribution of the random error is considered to be a model misspecification. Likewise, the causal parameter (e.g., $\:{\beta\:}_{1}$ in Eq. 4) and the dose-response function (e.g., $\:{\beta\:}_{1}t$ for $\:t\in\:\mathcal{T}=\left[{t}_{min},{t}_{max}\right]$ in Eq. 4) can be identified by fitting a weighted univariate linear model of the outcome *Y* on the exposure *T* based on observed data. In practical applications, flexible models, such as spline regression and local linear regression, can be used to estimate a nonlinear dose-response function. Here, a linear model is used for better clarity of the idea of IPW.

### NpmvCBGPS

The classical IPW method requires that the exposure model is adequately specified. As model misspecification is difficult to diagnose and assess, several works have focused on directly estimating IPW weights based on the fact that the correlation between the continuous exposure and covariates is the source of confounding bias [33]. Most of these methods build optimization criteria with the goal of covariate balance. Covariate balance means independence between the exposure and covariates after weighting [33]. One of the metrics commonly used to quantify covariate balance is that the weighted cross-moments between the exposure and covariates are zero. The nonparametric covariate balancing generalized propensity score (npCBGPS) method is one of these methods and is attractive because of its good performance in estimation and simple implementation [23]. In this study, we extend the npCBGPS to multiple continuous exposures. The input of the npmvCBGPS includes *m* continuous exposures $\:{\varvec{T}}_{\varvec{i}}=\left({T}_{1},\dots\:.{,T}_{m}\right)$ (e.g., *m* = 4 PFASs as stated in the real data analysis), *p* covariates $\:{\varvec{X}}_{\varvec{i}}=\left({X}_{1},\dots\:.,{X}_{p}\right)$ (e.g., *p* = 8 covariates as stated in the real data analysis), and an outcome $\:{Y}_{i}$ (e.g., BMI as stated in the real data analysis), *i=1*,*2*,*…,n*. We begin by centering and orthogonalizing the exposures and covariates as follows, which are only used to estimate IPW weights:

$$\:{\varvec{T}}_{i}^{\text{*}}={\varvec{S}}_{T}^{-1/2}\left({\varvec{T}}_{i}-\stackrel{-}{\varvec{T}}\right)$$

where $\:\stackrel{-}{\varvec{T}}={\sum\:}_{i=1}^{n}{\varvec{T}}_{i}/n$ and $\:{\varvec{S}}_{\varvec{T}}=\left({\varvec{T}}_{i}-\stackrel{-}{\varvec{T}}\right){\left({\varvec{T}}_{i}-\stackrel{-}{\varvec{T}}\right)}^{{\prime\:}}/\left(n-1\right)$ are the sample mean vector and $\:m\times\:m$ sample covariance matrix of ***T***, respectively; $\:{\varvec{S}}_{T}^{-1/2}$ is the inverse square root of $\:{\varvec{S}}_{\varvec{T}}$. Similarly, we transform the covariates:

$$\:{\varvec{X}}_{i}^{\text{*}}={\varvec{S}}_{X}^{-1/2}\left({\varvec{X}}_{i}-\stackrel{-}{\varvec{X}}\right)$$

where $\:\stackrel{-}{\varvec{X}}={\sum\:}_{i=1}^{n}{\varvec{X}}_{i}/n$ and $\:{\varvec{S}}_{\varvec{X}}=\left({\varvec{X}}_{i}-\stackrel{-}{\varvec{X}}\right){\left({\varvec{X}}_{i}-\stackrel{-}{\varvec{X}}\right)}^{{\prime\:}}/\left(n-1\right)$ are the sample mean vector and $\:p\times\:p$ sample covariance matrix of ***X***, respectively; $\:{\varvec{S}}_{X}^{-1/2}$ is the inverse square root of $\:{\varvec{S}}_{\varvec{X}}$. There are no correlations among the transformed covariates and among the transformed exposures. Then, the stabilizing weights can be defined as:

$${w}_{i}=\frac{f\left({\varvec{T}}_{\varvec{i}}^{\text{*}}\right)}{f\left({\varvec{T}}_{i}^{\text{*}}|{\varvec{X}}_{i}^{\text{*}}\right)}$$

The npmvCBGPS uses an empirical likelihood approach to choose weights that meet balancing conditions. The joint density of the *i*th individual in relation to the weights can be expressed as:

$$\begin{aligned}f\left({\varvec{T}}_{i}^{\text{*}},{\varvec{X}}_{i}^{\text{*}}\right)&=f\left({T}_{i}^{\text{*}}|{\varvec{X}}_{i}^{\text{*}}\right)f\left({\varvec{X}}_{i}^{\text{*}}\right)f\left({\varvec{T}}_{i}^{\text{*}}\right)\frac{1}{f\left({\varvec{T}}_{i}^{\text{*}}\right)}\\&=\frac{f\left({\varvec{T}}_{\varvec{i}}^{\text{*}}|{\varvec{X}}_{i}^{\text{*}}\right)}{f\left({T}_{i}^{\text{*}}\right)}f\left({\varvec{X}}_{i}^{\text{*}}\right)f\left({\varvec{T}}_{\varvec{i}}^{\text{*}}\right)\\&=\frac{1}{{w}_{i}}f\left({\varvec{X}}_{i}^{\text{*}}\right)f\left({\varvec{T}}_{\varvec{i}}^{\text{*}}\right)\end{aligned}$$

Thus, the likelihood function for the whole sample is expressed as:

6 $$\prod\:_{i=1}^{n}f\left({\varvec{T}}_{i}^{\text{*}},{\varvec{X}}_{i}^{\text{*}}\right)=\prod\:_{i=1}^{n}\frac{1}{{w}_{i}}f\left({\varvec{X}}_{i}^{\text{*}}\right)f\left({\varvec{T}}_{\varvec{i}}^{\text{*}}\right)$$

We now choose weights $\:{w}_{i}$ by maximizing Eq. (6), but also require $\:{w}_{i}$ to satisfy the following constraints [23]:

- (A1) $\:\mathbb{E}\left({w}_{i}{\varvec{T}}_{\varvec{i}}^{\text{*}}{\varvec{X}}_{\varvec{i}}^{\text{*}{\prime\:}}\right)=0$, which means that $\:{\varvec{T}}_{i}^{\text{*}}$ and $\:{\varvec{X}}_{\varvec{i}}^{\text{*}}$ are uncorrelated after weighting (the original $\:{\varvec{T}}_{\varvec{i}}$ and $\:{\varvec{X}}_{i}$ are also uncorrelated). This condition guarantees that covariates are balanced across different exposure levels after weighting.
- (A2) $\:\mathbb{E}\left({w}_{i}{\varvec{T}}_{\varvec{i}}^{\text{*}}\right)=0$ and $\:\mathbb{E}\left({w}_{i}{\varvec{X}}_{\varvec{i}}^{\text{*}}\right)=0$, which mean the marginal means of $\:{\varvec{T}}_{i}^{\text{*}}$ and $\:{\varvec{X}}_{\varvec{i}}^{\text{*}}$ are preserved after weighting.
- (A3) $\:\mathbb{E}\left({w}_{i}\right)=1$ and $\:{w}_{i}>0$, which mean weights are positive and they sum to the sample size *n*.

After log transformation of Eq. (6), the above is equivalent to maximizing:

$$\:{\sum\:}_{i=1}^{n}logf\left({\varvec{T}}_{\varvec{i}}^{\text{*}}\right)+logf\left({\varvec{X}}_{i}^{\text{*}}\right)-log{w}_{i}$$

subject to the above constraints. Because there are no correlations among the transformed covariates $\:{\varvec{X}}_{i}^{*}$ and among the transformed exposures $\:{\varvec{T}}_{i}^{*}$, the estimation of the npmvCBGPS is simplified to finding:

$$\:\underset{w\in\:{\mathbb{R}}^{n}}{\text{argmin}}{\sum\:}_{i=1}^{n}log{w}_{i}$$

subject to the above constraints. The details about solving this optimization problem can be found in the study by Fong et al. [23].

Under the causal identification assumptions, we can identify causal parameters or a dose-response function by fitting a weighted model in which the original outcome *Y* is regressed on the original exposure ***T*** [20]. For example, a weighted multiple linear regression model is as follows:

7 $$\:E\left[Y|\varvec{T}\right]={\beta\:}_{0}+{\sum\:}_{k=1}^{m}{\beta\:}_{k}{T}_{k}$$

### Simulation studies

#### Simulation setup

**Fig. 1:**
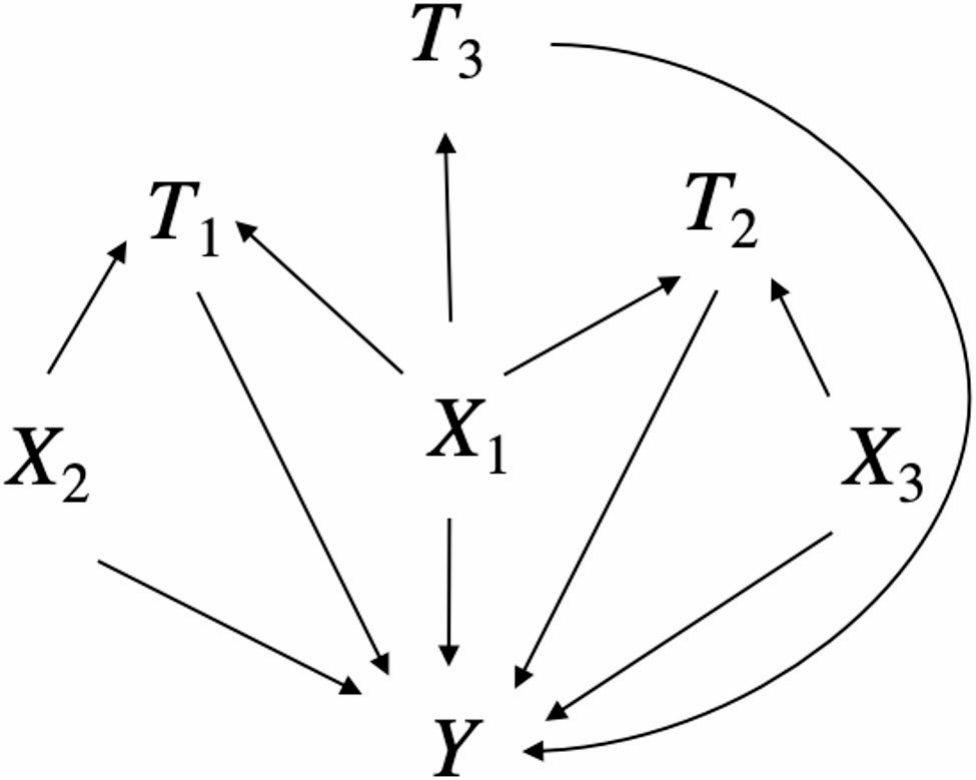
Directed acyclic graphs depicting three confounders

To assess the finite sample properties of the npmvCBGPS and compare it with the mvGPS method and the typical linear regression model, we conducted simulation studies under two scenarios. These simulations were designed to evaluate: (1) the accuracy and precision of causal estimates under Scenario 1, where both the exposure and outcome models were linear in covariates; (2) robustness to model misspecification under Scenario 2, where either the exposure or the outcome model was nonlinear in covariates. Additionally, we included a simple interaction among exposures in the outcome model across both scenarios to investigate the impact of nonlinear exposure effects. We modified the simulation studies of Fong et al. and Williams et al. to align with real-world environmental health studies (where environmental mixtures typically comprise three or more exposures with effects in different directions), while maintaining parsimony to elucidate the strengths and limitations of estimators [7, 20, 23]. Therefore, we considered three exposures, a continuous outcome, and three confounders that reflect different degrees of overlap in confounding for the exposures as illustrated in Fig. 1 (common confounding: $\:{X}_{1}$; partially common confounding: $\:{X}_{2}$ and $\:{X}_{3}$) [20]. Covariates that only predict exposures ($\:{X}_{4}$, $\:{X}_{5}$, $\:{X}_{6}$) were also considered because they may affect the variance of an estimate [34].

The first step of the simulation was to generate 6 covariates. Five covariates were drawn from a multivariate normal distribution, $\:\varvec{X}=\left({X}_{1},{X}_{3},{X}_{4},{X}_{5},{X}_{6}\right)\sim{N}_{5}\left(0,\sum\:\right)$, with means of 0, variances of **1**, and covariances of 0.2. A binary covariate $\:{X}_{2}$ was generated from a Bernoulli distribution, ${X}_{2}{\sim}Bernoulli\;({p}_{2}=0.5)$. The data-generating processes have been summarized in supplementary Table S1.

#### Scenario 1

The data-generating processes in Scenario 1 were as follows:

- **(A) E1Y1 and E1Y2**: exposures were generated by:

8 $$\:\text{E}\text{x}\text{p}\text{o}\text{s}\text{u}\text{r}\text{e}\:\text{m}\text{o}\text{d}\text{e}\text{l}\:\text{E}1:{\varvec{T}}_{i}={\varvec{X}}_{i}{\varvec{\alpha\:}}_{1}+{\varsigma\:}_{i}\:{\varvec{\alpha\:}}_{1}=\left(\begin{array}{c}\text{1,1},\text{0,1},\text{1,0}\\\:\text{1,0},\text{1,1},\text{0,1}\\\:1,\text{0,0},\text{0,0},0\end{array}\right)$$

where $\:\varsigma\:$ is the random error simulated from a multivariate normal distribution, $\:\varsigma\:\sim{N}_{3}\left(0,\varvec{M}\right)$, $$\:\varvec{M}=\left(\begin{array}{ccc}1&\:Trho&\:Trho\\\:Trho&\:1&\:Trho\\\:Trho&\:Trho&\:1\end{array}\right)$$ with means of 0, variances of 1. We set the covariance element $\:Trho$ to 0.2, 0.5, and 0.7 to explore the impact of collinearity among mixture components, which is a core challenge in mixture analysis [12].

The outcome was generated by:

$$\begin{aligned}\text{Outcome model Y1}:\,{Y}_{i}=&{0.6T}_{i1}-0.5{T}_{i2}+{0.8T}_{i3}\\&+{\varvec{X}}_{\varvec{i}}{\varvec{\beta\:}}_{1}+{\zeta\:}_{i}\end{aligned}$$

$$\begin{aligned}\text{Outcome model Y2}:{Y}_{i}=&{0.6T}_{i1}-0.5{T}_{i2}+{0.8T}_{i3}\\&+0.2{T}_{i1}{T}_{i3}+{\varvec{X}}_{\varvec{i}}{\varvec{\beta\:}}_{1}+{\zeta}_{i}\end{aligned}$$

$${\varvec{\beta\:}}_{1}=\left(\text{1,1},\text{1,0},\text{0,0}\right)$$

where $\:\zeta\:$ is the random error simulated from a standard normal distribution, $\zeta\:{\sim}N\left(\text{0,1}\right)$, with a mean of 0 and a variance of 1. We set the exposure effects to 0.2, −0.5, 0.6, and 0.8 because they are commonly found in epidemiology [35].

#### Scenario 2

In Scenario 2, we generated true data under the following settings:

- **(B) E2Y1 and E2Y2**: the outcome model was correctly specified, whereas the exposure model was misspecified.

9 $$\text{Exposure model E2}:{\varvec{T}}_{\varvec{i}}={\varvec{X}}_{i}{\varvec{\alpha\:}}_{1}+\left({\varvec{X}}_{i}\text{*}{\varvec{X}}_{i}\right){\varvec{\alpha\:}}_{2}+{\varsigma\:}_{i}$$

$$\begin{array}{cc}{\varvec{\alpha}}_1=\begin{pmatrix}\text{1,1},\text{0,1},\text{1,0}\\\:\text{1,0},\text{1,1},\text{0,1}\\\:1,\text{0,0},\text{0,0},0\end{pmatrix}&{\varvec{\alpha}}_{2}=\left(\begin{array}{c}\begin{array}{c}\text{1,0},\text{0,0},\text{0,0}\\\:\text{1,0},\text{0,0},\text{0,0}\end{array}\\\:\text{1,0},\text{0,0},\text{0,0}\end{array}\right)\end{array}$$

where $\:\varsigma\:\sim{N}_{3}\left(0,\varvec{M}\right)$ with means of 0, variances of 1, and covariances of 0.2, 0.5, and 0.7. The outcome model remained the same as in Scenario 1.

- **(C) E1Y3 and E1Y4**: the outcome model was misspecified, whereas the exposure model was correctly specified.

$$\begin{aligned}\text{Outcome model Y3}:{Y}_{i}=&{0.6T}_{i1}-0.5{T}_{i2}+{0.8T}_{i3}\\&+{\varvec{X}}_{i}{\varvec{\beta\:}}_{1}+\left({\varvec{X}}_{i}\text{*}{\varvec{X}}_{i}\right){\varvec{\beta\:}}_{2}+{\zeta\:}_{i}\end{aligned}$$

$$\begin{aligned}\text{Outcome model Y4}:{Y}_{i}=&{0.6T}_{i1}-0.5{T}_{i2}+{0.8T}_{i3}\\&+0.2{T}_{i1}{T}_{i3}+{\varvec{X}}_{i}{\varvec{\beta\:}}_{1}\\&+\left({\varvec{X}}_{i}\text{*}{\varvec{X}}_{i}\right){\varvec{\beta\:}}_{2}+{\zeta\:}_{i}\end{aligned}$$

where $\zeta\:{\sim}N\left(\text{0,1}\right)$ and $\:{\varvec{\beta\:}}_{1}=\left(\text{1,1},1,\text{0,0},0\right)$, $\:{\varvec{\beta\:}}_{2}=\left(\text{1,0},\text{0,0},\text{0,0}\right)$. The exposure model remained the same as in Scenario 1.

- **(D) E2Y3 and E2Y4**: both the exposure and outcome models were misspecified.

We simulated 500 datasets for each setting with sample sizes of 200, 500, and 1000 and reported statistics related to causal parameters (coefficients of exposures): bias that evaluates the accuracy of estimates (the mean of estimates minus the true value); the root mean squared error (RMSE) that evaluates the precision of estimates, the smaller the RMSE, the higher the precision; the 95% CI coverage (the proportion of which estimated CIs include the true value); power (the proportion that hypothesis test with *P* < 0.05). Additionally, the average absolute weighted Pearson correlations between exposures and confounders were used to assess covariate balance, and a value of 0.1 has been suggested to be acceptable in practice [20, 36]. Covariate balance is crucial, as imbalances may result in biased estimates [23]. We developed the R package mvCBGPS available at https://github.com/QianGao-SXMU/mvCBGPS and Supplementary data to implement the npmvCBGPS method.

We compared the proposed npmvCBGPS method with the following two methods: (1) the linear regression model for the outcome that controls for all confounders; (2) mvGPS, which fits the exposure model using a multiple multivariate linear model and subsequently assumes a multivariate normal distribution for the random error to calculate balance weights.

### Per- and polyfluoroalkyl substances and BMI

To test npmvCBGPS and mvGPS on real data, we used publicly available NHANES data to associate Perfluoroalkyl and Polyfluoroalkyl Substances (PFASs) with body mass index (BMI). Several epidemiologic studies of associations between PFASs with BMI have reported mixed findings, including both positive and no effects in adults [37]. The National Health and Nutrition Examination Survey (NHANES) is a nationally representative survey that measures the health and nutritional status of adults and children in the US every 2 years. The US Centers for Disease Control and Prevention has described the study procedures of NHANES in detail [38]. This study pooled cross-sectional data from the NHANES 2003 — 2018 cycles. Survey participants who met the following criteria were included: (1) aged 20 years and older; (2) with complete data on concentrations of PFASs in serum and covariates. Participants who had a positive pregnancy test at the time of the survey were excluded [14]. A total of 12,714 participants were included in the final analysis (Figure S1). NHANES received ethical approval from the National Center for Health Statistics. Written consent was obtained from all participants before their engagement in NHANES.

PFASs with a detection rate below 80% were not included in this study [13], leaving perfluorooctanoic acid (PFOA), perfluorooctane sulfonic acid (PFOS), perfluorohexane sulfonic acid (PFHxS), and perfluorononanoic acid (PFNA) for the analysis. The concentrations of PFASs were naturally log-transformed [13, 14]. There were eight covariates, including age, gender, race or ethnicity, highest educational level, marital status, cigarette smoking (based on serum cotinine level), income-to-poverty ratio, and total energy intake. We explored the linear and nonlinear relationships between PFASs and BMI using the IPW method.

## Results

### Simulation results

#### Covariate balance and Estimation under scenario 1 and scenario 2

**Fig. 2:**
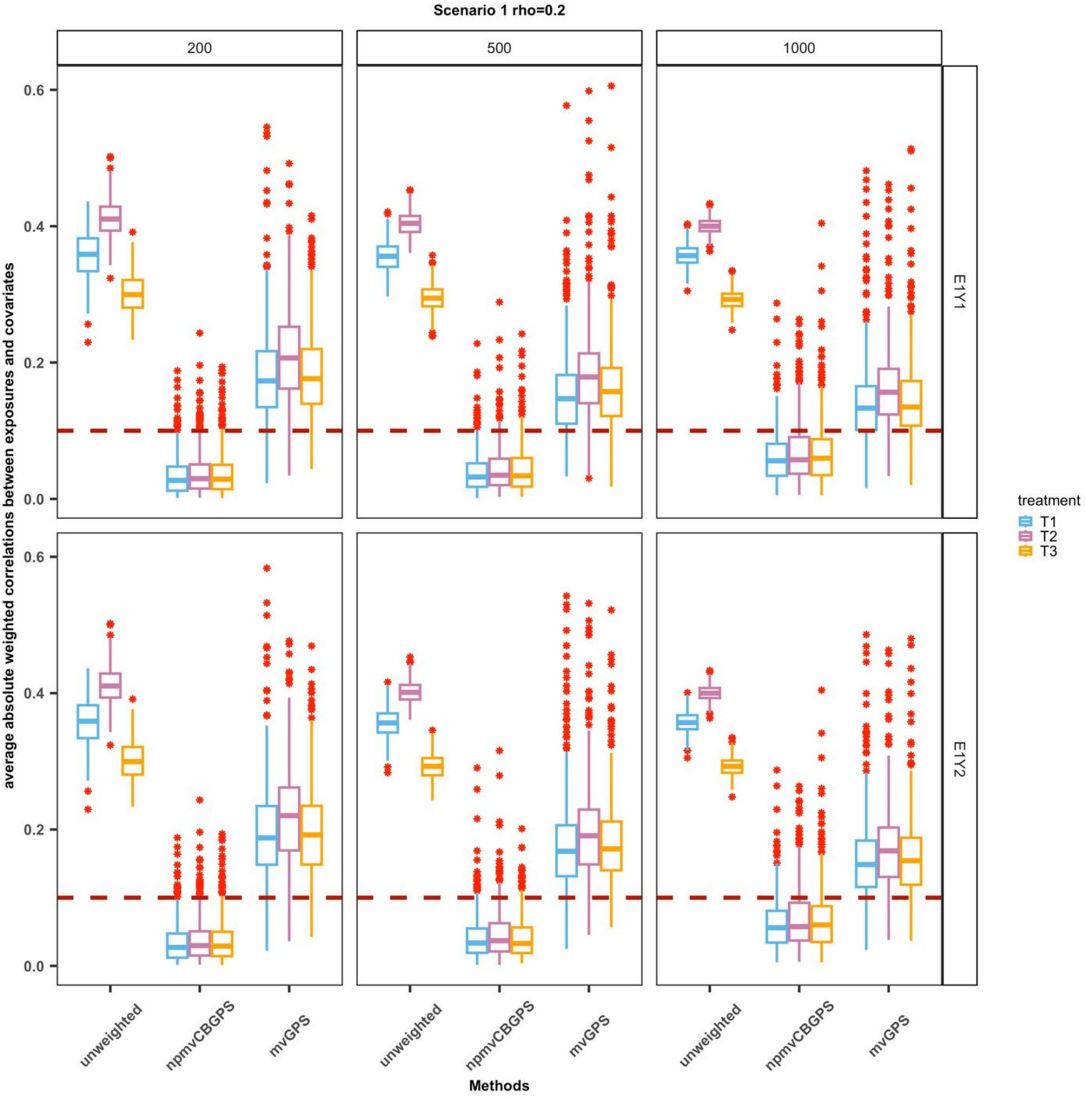
The performance of covariate balance under Scenario 1

In Scenario 1, we compared the accuracy and precision of the proposed npmvCBGPS, mvGPS method, and the linear regression model for the outcome. Figure 2 illustrates the degree of covariate balance achieved by the two GPS methods along with the original unweighted correlations for comparison. In general, both the npmvCBGPS and mvGPS methods improved the covariate balance compared with the original sample in all settings. The mean of weighted correlations for the npmvCBGPS method was less than 0.1, whereas that for the mvGPS method was greater than 0.1. Table 1 shows the summary statistics of the causal parameter estimates under Scenario 1. When both the exposure and outcome models were linear in the covariates and exposures (E1Y1), npmvCBGPS yielded causal parameter estimates with biases that were close to 0 for all three exposures. As the sample size increased, both bias and RMSE tended to decrease for the npmvCBGPS. The mean biases and RMSEs of mvGPS were higher than those of npmvCBGPS. When there was an interaction among three-dimensional exposures (E1Y2), the results for the main effects of the three exposures were similar to those in E1Y1, that is, npmvCBGPS outperformed mvGPS in terms of mean biases and RMSEs. For the interaction effect, the mean biases and RMSEs were smaller than those of the main effects. As expected, in both settings, the linear regression model yielded biases that were close to those of npmvCBGPS, but with higher precision.

**Table 1:** Mean bias and RMSE of the causal parameter estimates under Scenario 1

Settings	Method	n	T1 (0.6)		T2 (−0.5)		T3 (0.8)		T1T3 (0.2)	
			bias	RMSE	bias	RMSE	bias	RMSE	bias	RMSE
E1Y1	npmvCBGPS	200	0.006	0.126	0.022	0.121	0.045	0.187	-	-
		500	0.006	0.096	0.013	0.094	0.033	0.154		
		1000	0.001	0.099	0.028	0.099	0.058	0.161		
	mvGPS	200	0.033	0.172	0.126	0.206	0.154	0.278	-	-
		500	0.026	0.148	0.103	0.178	0.122	0.258		
		1000	0.022	0.134	0.090	0.145	0.119	0.225		
	linear	200	−0.002	0.076	0.006	0.079	−0.001	0.078	-	-
		500	−0.001	0.046	0.000	0.044	−0.002	0.048		
		1000	0.002	0.031	0.000	0.034	−0.003	0.032		
E1Y2	npmvCBGPS	200	0.008	0.127	0.025	0.121	0.048	0.195	−0.001	0.109
		500	0.013	0.092	0.015	0.094	0.040	0.158	−0.003	0.081
		1000	0.006	0.094	0.030	0.098	0.062	0.158	−0.003	0.073
	mvGPS	200	0.040	0.168	0.129	0.205	0.182	0.293	−0.030	0.098
		500	0.038	0.158	0.104	0.174	0.150	0.239	−0.021	0.070
		1000	0.026	0.129	0.095	0.144	0.147	0.232	−0.022	0.066
	linear	200	−0.002	0.076	0.006	0.079	0.000	0.079	0.001	0.021
		500	−0.002	0.048	−0.002	0.046	−0.001	0.048	0.000	0.012
		1000	0.002	0.031	0.000	0.034	−0.003	0.032	0.000	0.009

**Fig. 3:**
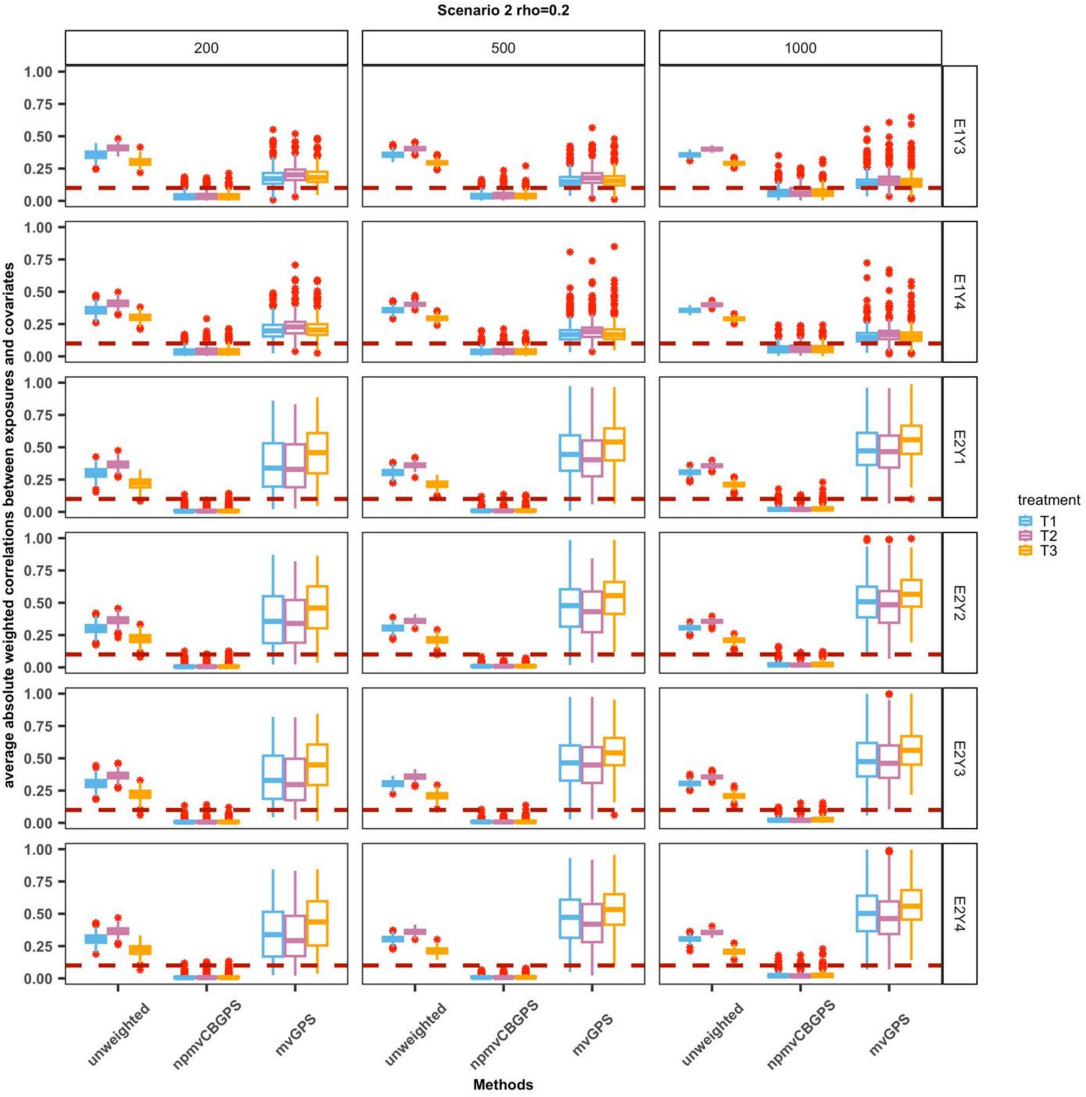
The performance of covariate balance under Scenario 2

In Scenario 2, we assessed the robustness to model misspecification of the npmvCBGPS, mvGPS, and the linear regression model. Figure 3 illustrates the degree of covariate balance in Scenario 2. In general, the npmvCBGPS method improved the covariate balance, with mean weighted correlations of less than 0.1 in all settings. Compared with the original sample, the covariate balance was improved for the mvGPS method when only the exposure model was correctly specified (E1Y3 and E1Y4), whereas when the exposure model was misspecified, the mvGPS exhibited limited balance improvement. The summary statistics of the causal parameter estimates under Scenario 2 are listed in Table 2. When only the outcome model was incorrectly specified (E1Y3 and E1Y4), results for the npmvCBGPS and mvGPS were similar to those in Scenario 1, that is, npmvCBGPS outperformed mvGPS in terms of mean biases and RMSEs, whereas the accuracy and precision decreased for the linear regression model, especially when there was an interaction among three-dimensional exposures (E1Y4). When only the exposure model was incorrectly specified (E2Y1 and E2Y2), npmvCBGPS and the linear regression model still provided estimates with mean biases that were close to 0, whereas mvGPS provided estimates with mean biases that were more likely to deviate from 0, and the RMSEs significantly increased. Not surprisingly, npmvCBGPS, mvGPS, and the linear regression model failed when neither the exposure model nor the outcome model was correctly specified (E2Y3 and E2Y4).

**Table 2:** Mean bias and RMSE of the causal parameter estimates under Scenario 2

Settings	Method	n	T1 (0.6)		T2 (−0.5)		T3 (0.8)		T1T3 (0.2)	
			bias	RMSE	bias	RMSE	bias	RMSE	bias	RMSE
E1Y3	npmvCBGPS	200	0.015	0.128	0.021	0.132	0.027	0.199	-	-
		500	0.005	0.100	0.016	0.109	0.020	0.162		
		1000	0.002	0.115	0.030	0.115	0.055	0.184		
	mvGPS	200	0.023	0.213	0.121	0.214	0.175	0.355	-	-
		500	0.025	0.159	0.110	0.181	0.117	0.264		
		1000	0.021	0.168	0.075	0.169	0.125	0.255		
	linear	200	0.002	0.123	−0.005	0.128	−0.007	0.140	-	-
		500	−0.004	0.081	0.004	0.082	−0.006	0.078		
		1000	0.002	0.055	0.001	0.057	0.001	0.058		
E1Y4	npmvCBGPS	200	0.008	0.140	0.022	0.130	0.006	0.227	0.064	0.135
		500	0.013	0.104	0.011	0.105	−0.006	0.166	0.084	0.121
		1000	0.011	0.100	0.022	0.111	0.011	0.165	0.088	0.117
	mvGPS	200	0.038	0.220	0.128	0.222	0.178	0.343	0.016	0.108
		500	0.030	0.186	0.109	0.210	0.142	0.278	0.001	0.087
		1000	0.037	0.153	0.090	0.170	0.126	0.264	−0.015	0.076
	linear	200	−0.002	0.123	0.001	0.115	−0.108	0.167	0.205	0.210
		500	−0.001	0.076	0.001	0.074	−0.105	0.133	0.208	0.210
		1000	0.000	0.051	0.004	0.052	−0.103	0.119	0.208	0.209
E2Y1	npmvCBGPS	200	0.001	0.086	−0.002	0.088	0.010	0.218	-	-
		500	−0.002	0.068	0.001	0.069	0.004	0.211		
		1000	0.003	0.055	0.002	0.060	0.005	0.209		
	mvGPS	200	−0.018	0.253	0.108	0.286	−0.504	0.606	-	-
		500	−0.040	0.401	0.105	0.325	−0.539	0.674		
		1000	−0.080	0.561	0.110	0.453	−0.442	0.620		
	linear	200	−0.004	0.067	−0.001	0.065	0.002	0.065	-	-
		500	0.002	0.042	0.001	0.045	−0.001	0.042		
		1000	0.000	0.029	0.003	0.030	−0.002	0.030		
E2Y2	npmvCBGPS	200	0.028	0.109	0.007	0.088	0.061	0.199	−0.016	0.052
		500	0.035	0.089	0.012	0.069	0.104	0.179	−0.026	0.045
		1000	0.054	0.084	0.016	0.064	0.140	0.196	−0.037	0.047
	mvGPS	200	−0.062	0.277	0.132	0.277	−0.481	0.619	0.017	0.102
		500	−0.087	0.337	0.110	0.279	−0.607	0.728	0.020	0.095
		1000	−0.192	0.482	0.112	0.362	−0.613	0.777	0.033	0.095
	linear	200	0.002	0.069	−0.002	0.065	−0.004	0.074	0.000	0.010
		500	0.001	0.045	0.004	0.045	−0.003	0.043	0.000	0.006
		1000	−0.003	0.032	0.001	0.030	−0.001	0.031	0.000	0.004
E2Y3	npmvCBGPS	200	0.079	0.139	0.077	0.134	0.523	0.542	-	-
		500	0.092	0.123	0.085	0.119	0.553	0.563		
		1000	0.096	0.119	0.086	0.113	0.574	0.581		
	mvGPS	200	0.096	0.282	0.162	0.270	0.529	0.602	-	-
		500	0.081	0.347	0.180	0.346	0.621	0.713		
		1000	0.141	0.486	0.172	0.452	0.586	0.733		
	linear	200	0.270	0.282	0.261	0.273	0.268	0.278	-	-
		500	0.267	0.271	0.270	0.274	0.265	0.270		
		1000	0.270	0.272	0.272	0.274	0.269	0.271		
E2Y4	npmvCBGPS	200	0.027	0.119	0.069	0.131	0.363	0.400	0.055	0.066
		500	0.038	0.090	0.068	0.100	0.411	0.428	0.046	0.052
		1000	0.047	0.084	0.072	0.100	0.428	0.439	0.042	0.047
	mvGPS	200	−0.011	0.204	0.164	0.240	0.319	0.468	0.069	0.106
		500	−0.022	0.286	0.175	0.308	0.401	0.587	0.052	0.098
		1000	0.012	0.458	0.211	0.512	0.425	0.709	0.038	0.118
	linear	200	0.199	0.215	0.232	0.246	0.182	0.203	0.031	0.034
		500	0.209	0.215	0.231	0.237	0.192	0.200	0.027	0.028
		1000	0.211	0.214	0.232	0.235	0.196	0.200	0.026	0.027

#### Statistical testing under scenario 1 and scenario 2

Table 3 shows the results of 95% CI coverage and power under Scenario 1. The npmvCBGPS provided power greater than 92.8% and 95% CI coverage close to the nominal value of 95% in all cases except when the sample size was 1000 (87.2% − 94.8%). Compared with npmvCBGPS, mvGPS had slightly reduced power (86.2% − 98.8%), but poor 95% CI coverage (58.2% − 87.8%). As expected, the linear regression model was more powerful with power of 1 and provided 95% CI coverage that was close to the nominal value in all cases.

**Table 3:** Confidence intervals and power of the causal parameter estimates under Scenario 1

Settings	Method	n	T1(0.6)		T2(−0.5)	T3(0.8)		
			coverage	power	coverage	power	coverage	power
E1Y1	npmvCBGPS	200	0.938	0.968	0.942	0.928	0.942	0.942
		500	0.968	0.994	0.950	0.992	0.952	0.980
		1000	0.948	0.986	0.902	0.994	0.880	0.982
	mvGPS	200	0.876	0.956	0.662	0.864	0.760	0.966
		500	0.878	0.988	0.582	0.946	0.718	0.966
		1000	0.864	0.974	0.614	0.976	0.636	0.982
	linear	200	0.944	1	0.948	1	0.944	1
		500	0.964	1	0.952	1	0.946	1
		1000	0.960	1	0.952	1	0.964	1
E1Y2	npmvCBGPS	200	0.930	0.970	0.930	0.936	0.936	0.944
		500	0.978	0.994	0.922	0.996	0.926	0.990
		1000	0.948	0.990	0.884	0.994	0.872	0.994
	mvGPS	200	0.866	0.966	0.638	0.862	0.746	0.970
		500	0.862	0.978	0.606	0.946	0.684	0.982
		1000	0.862	0.982	0.592	0.98	0.620	0.986
	linear	200	0.95	1	0.948	1	0.940	1
		500	0.944	1	0.968	1	0.952	1
		1000	0.966	1	0.952	1	0.964	1

Table 4. shows the results of statistical testing under Scenario 2. When only the outcome model was misspecified (E1Y3 and E1Y4), the results for npmvCBGPS and mvGPS were similar to those in Scenario 1. In contrast, the power was slightly reduced, and the 95% CI coverage was significantly decreased for the linear regression model, especially when there was an interaction among three-dimensional exposures (E1Y4). When only the exposure model was misspecified (E2Y1 and E2Y2), results for npmvCBGPS and the linear regression model were similar to those in Scenario 1, while mvGPS had significantly reduced coverage and power. When neither the exposure nor the outcome model was correctly specified (E2Y3 and E2Y4), both the linear regression model and npmvCBGPS were powerful but with significantly reduced 95%CI coverage.

**Table 4:** Confidence intervals and power of the causal parameter estimates under Scenario 2

Settings	Method	n	T1(0.6)		T2(−0.5)		T3(0.8)	
			coverage	power	coverage	power	coverage	power
E1Y3	npmvCBGPS	200	0.954	0.972	0.922	0.932	0.946	0.924
		500	0.970	0.986	0.942	0.970	0.952	0.970
		1000	0.932	0.976	0.878	0.986	0.860	0.956
	mvGPS	200	0.862	0.922	0.676	0.806	0.766	0.950
		500	0.884	0.960	0.650	0.912	0.770	0.962
		1000	0.896	0.952	0.680	0.942	0.766	0.968
	linear	200	0.958	0.998	0.960	0.972	0.918	1
		500	0.950	1	0.946	1	0.968	1
		1000	0.956	1	0.948	1	0.946	1
E1Y4	npmvCBGPS	200	0.940	0.956	0.952	0.934	0.924	0.890
		500	0.972	0.994	0.940	0.984	0.958	0.954
		1000	0.932	0.996	0.872	0.992	0.906	0.966
	mvGPS	200	0.862	0.938	0.694	0.794	0.772	0.936
		500	0.868	0.970	0.644	0.914	0.752	0.976
		1000	0.882	0.968	0.654	0.956	0.724	0.970
	linear	200	0.934	0.996	0.942	0.992	0.808	1
		500	0.938	1	0.944	1	0.690	1
		1000	0.952	1	0.954	1	0.494	1
E2Y1	npmvCBGPS	200	0.988	0.986	0.986	0.984	0.994	0.986
		500	0.982	0.996	0.980	1	1	1
		1000	0.994	1	0.980	1	0.998	0.998
	mvGPS	200	0.790	0.868	0.664	0.734	0.264	0.552
		500	0.674	0.812	0.628	0.748	0.200	0.618
		1000	0.556	0.748	0.550	0.694	0.260	0.662
	linear	200	0.950	1	0.964	1	0.958	1
		500	0.938	1	0.946	1	0.952	1
		1000	0.944	1	0.946	1	0.958	1
E2Y2	npmvCBGPS	200	0.960	0.992	0.988	0.986	0.910	0.982
		500	0.946	0.996	0.980	1	0.856	0.998
		1000	0.930	1	0.960	1	0.746	0.996
	mvGPS	200	0.842	0.806	0.640	0.736	0.468	0.466
		500	0.760	0.830	0.618	0.818	0.216	0.472
		1000	0.648	0.694	0.550	0.776	0.204	0.454
	linear	200	0.954	1	0.964	1	0.946	1
		500	0.952	1	0.920	1	0.960	1
		1000	0.946	1	0.944	1	0.950	1
E2Y3	npmvCBGPS	200	0.882	0.992	0.856	0.948	0.072	1
		500	0.750	1	0.768	0.992	0.002	1
		1000	0.650	1	0.722	1	0.002	1
	mvGPS	200	0.710	0.946	0.540	0.790	0.166	0.994
		500	0.592	0.902	0.518	0.698	0.096	0.990
		1000	0.554	0.888	0.454	0.668	0.166	0.978
	linear	200	0.070	1	0.078	0.836	0.072	1
		500	0.000	1	0.000	0.996	0.000	1
		1000	0.000	1	0.000	1	0.000	1
E2Y4	npmvCBGPS	200	0.916	0.992	0.840	0.966	0.344	1
		500	0.918	1	0.812	0.998	0.064	1
		1000	0.870	1	0.732	1	0.018	1
	mvGPS	200	0.824	0.938	0.508	0.816	0.416	0.966
		500	0.714	0.898	0.482	0.792	0.302	0.964
		1000	0.636	0.818	0.474	0.692	0.348	0.934
	linear	200	0.306	1	0.188	0.910	0.402	1
		500	0.010	1	0.004	1	0.038	1
		1000	0.000	1	0.000	1	0.002	1

#### Estimation under varying correlations among exposures

**Fig. 4:**
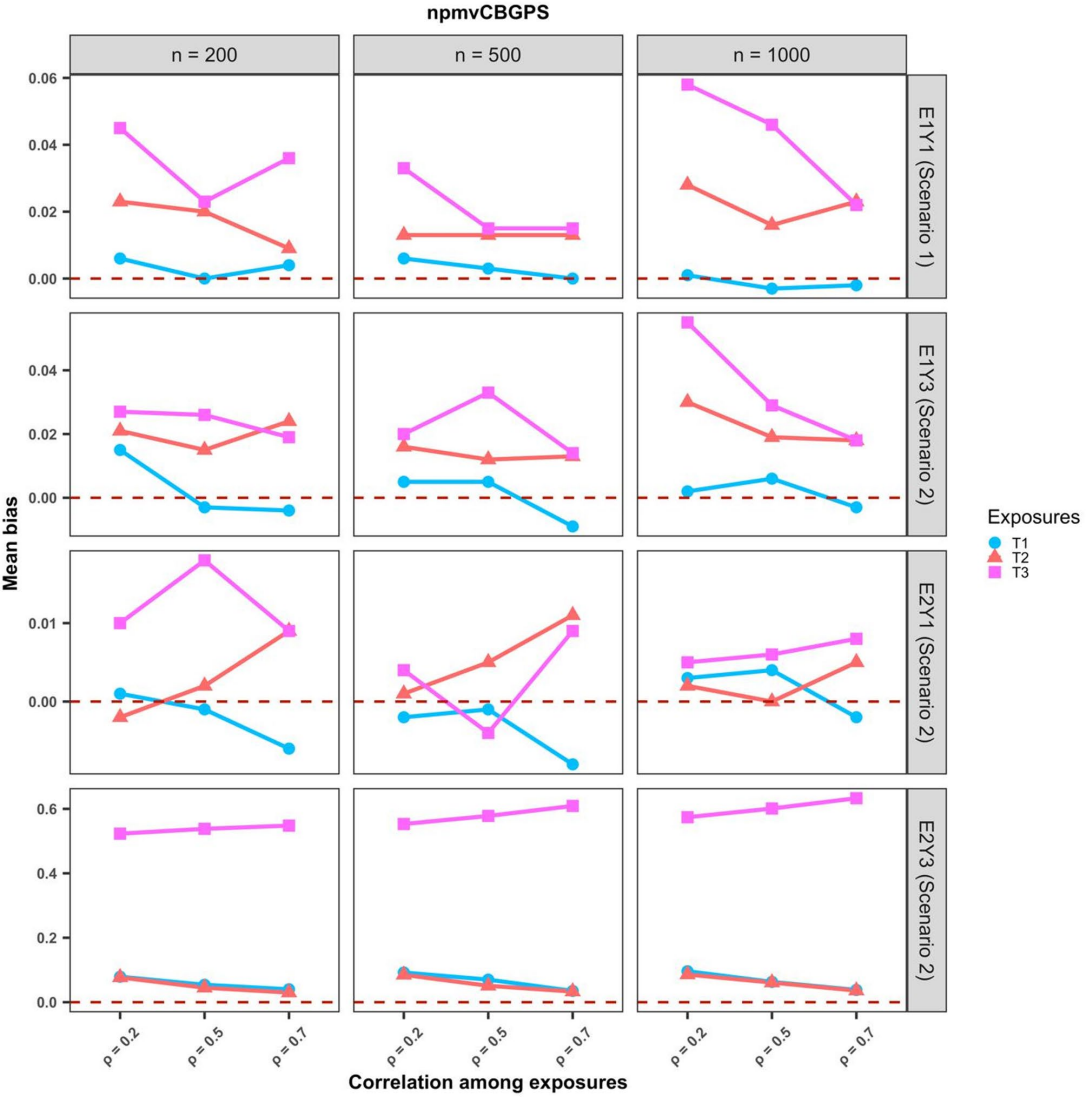
Mean biases of causal parameter estimates at varying exposure correlations for the npmvCBGPS method in cases where there was no interaction among exposures

**Fig. 5:**
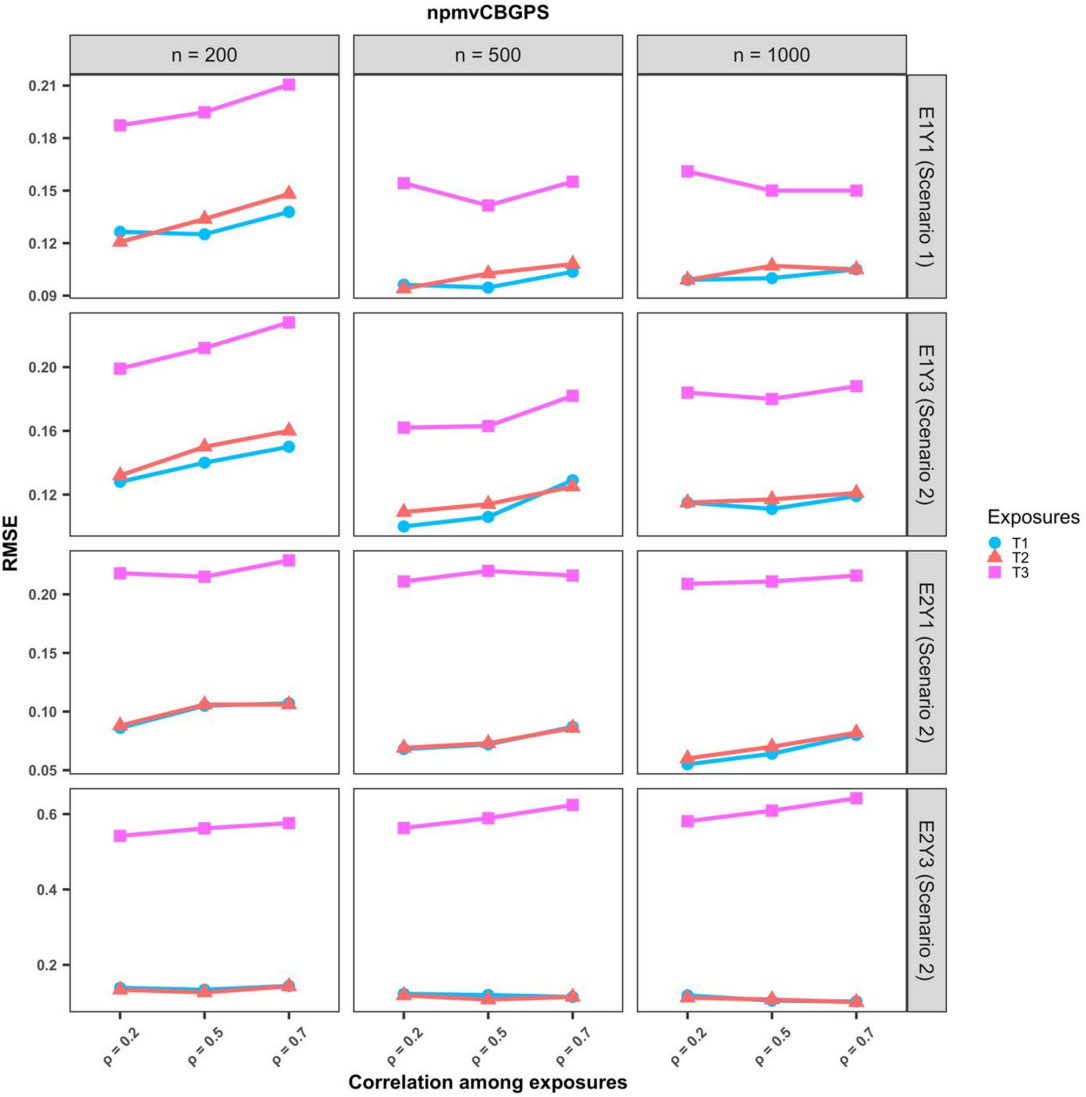
RMSE of causal parameter estimates at varying exposure correlations for the npmvCBGPS method in cases where there was no interaction among exposures

We repeated the simulations with varying correlations among exposures to explore the impact of collinearity. Figures 4 and 5 display the mean biases and RMSEs of causal parameter estimates at varying exposure correlations for the npmvCBGPS method, respectively, in cases where there was no interaction among exposures under two scenarios. The mean biases were less affected by the correlations among exposures (changes were less than 0.06). The RMSEs were slightly increased with increasing correlations among exposures (changes were less than 0.08). Results were similar when there was an interaction among exposures (Figures S2 and S3). The trend of mean biases and RMSEs with increasing correlations among exposures for the mvGPS method (Figures S4 – S7) and the linear regression model (Figures S8-S11) was similar to that of the npmvCBGPS method.

### Health effects of PFASs on BMI

**Fig. 6:**
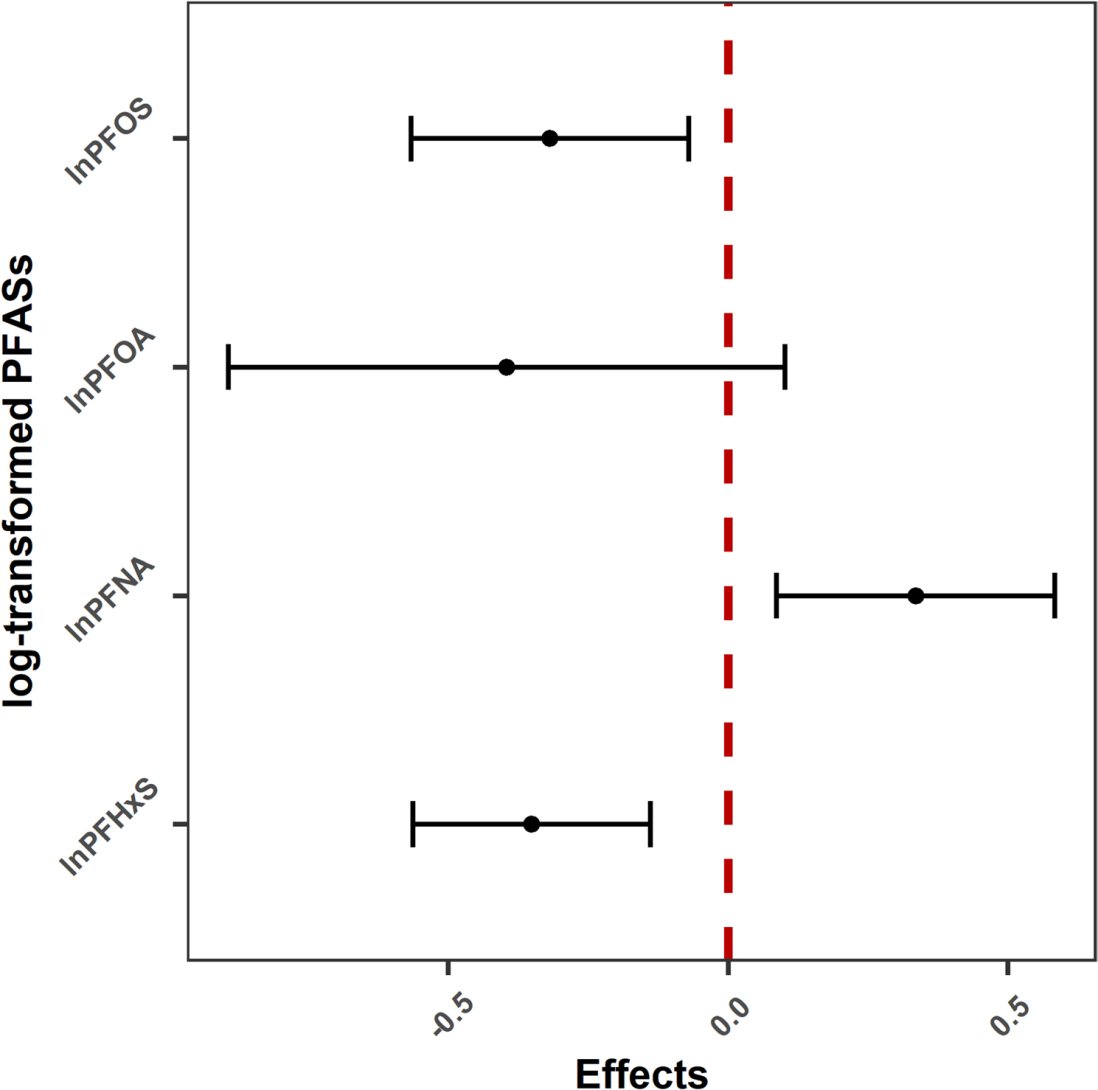
Forest plot for model coefficients evaluating the linear relationship between PFASs with BMI in the NHANES 2003-18

**Fig. 7:**
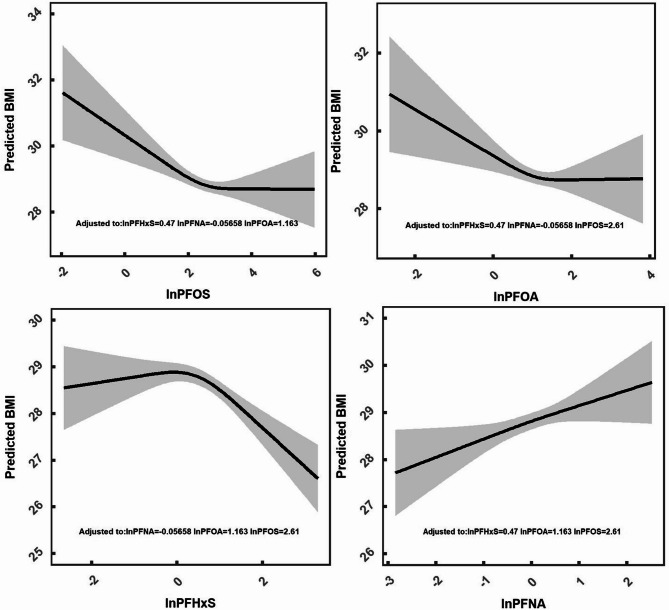
Single exposure-response functions of each PFAS component, where the remaining components are fixed at their median values

**Fig. 8:**
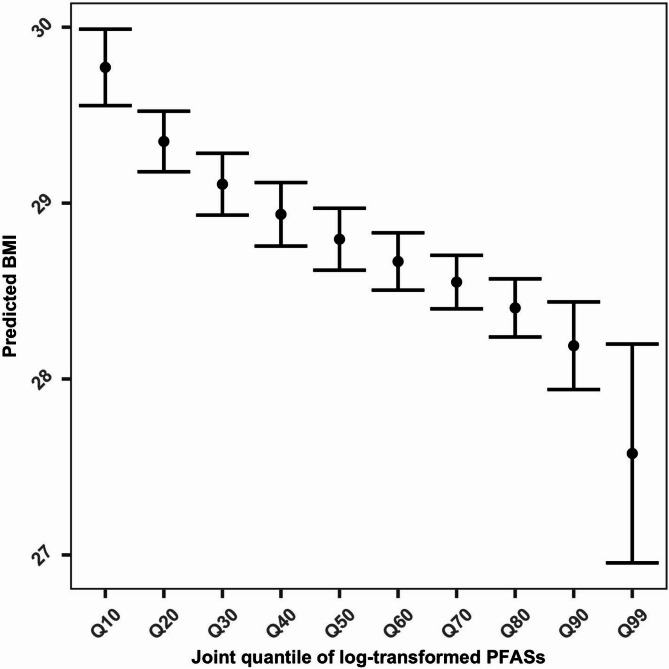
Joint exposure-response relationships and 95% confidence intervals (CIs) of PFASs on BMI

We first evaluated the covariate balance performance in the unweighted original and weighted samples. Results showed that covariate balance was improved compared with the original sample for npmvCBGPS (Figure S12), whereas there was a deterioration in balance after weighting for mvGPS (Figure S13). Therefore, we only show the results of npmvCBGPS. Model results that explored the linear association between PFASs and BMI are shown in Fig. 6. Numerical values for the coefficients displayed in this figure are shown in Table S2. There was a positive association between PFNA and BMI, whereas there were negative associations between PFOS, PFHxS with BMI. We used restricted cubic spline analysis to explore nonlinear associations. Figure 7 shows the single-exposure effect, which we defined as the dose-response curve of a single exposure on the outcome when all of the other exposures were fixed at their median values and all of the covariates were held constant [10]. We found nonlinear associations between PFOA, PFOS, PFHxS with BMI. Figure 8 shows the joint-exposure effect, which we defined as the dose-response association of mixed exposures on the outcome when all of the exposures were fixed at a given exposure percentile and the covariates were held constant. We observed an overall inverse trend between the PFAS mixtures with BMI.

## Discussion

Estimating the causal health effects of several concurrent environmental exposures yields promising results in inferring how interventions can improve public health [8]. To this end, we focused on a causal modeling approach for environmental exposure mixtures. We presented npmvCBGPS, an extension of GPS methods, which are a common statistical tool for estimating the causal effect of a single exposure in observational studies. Using simulations, we conducted an in-depth assessment of the statistical performance of npmvCBGPS and showed that npmvCBGPS provided estimates that were close to the true value as long as either the exposure model or the outcome model was correctly specified; that is, the results empirically demonstrated that npmvCBGPS had properties similar to doubly robust estimators. In contrast, the validity of the mvGPS and the linear regression model relied on the correct specification of the exposure model and the outcome model, respectively. The performance of the npmvCBGPS was less impacted by collinearity among mixture components. In addition, the npmvCBGPS outperformed the existing mvGPS method in terms of accuracy, precision, and statistical testing across all simulated settings.

The proposed npmvCBGPS advances causal modeling in mixture exposure analysis. The quantile g-computation and SL-Gcomp methods extended classical g-computation for multiple simultaneous continuous exposures [7, 24]. The quantile g-computation transformed each exposure into a categorical variable, which resulted in the loss of information, potentially compromising the substantive insights derived from data analysis [7, 23]. The SL-Gcomp method is robust to the outcome model misspecification but may be affected by the choice of tunning parameters and prediction algorithms [25, 26]. The existing mvGPS extended classical GPS methods for multiple simultaneous continuous exposures [20]. The mvGPS fitted a multiple multivariate linear model for exposures and subsequently assumed a multivariate normal distribution for exposures to calculate balancing weights. Consequently, the performance of mvGPS relied on the correct specification of the exposure model and distribution. This explains why the estimates of mvGPS start deviating from the true value when the exposure model was incorrectly specified (E2Y1 and E2Y2). Different from this work, npmvCBGPS estimated directly balancing weights without requiring the specification of the exposure model and distribution. As elaborated in simulation studies, the robustness of our estimator to exposure model misspecification was one of the main advantages over mvGPS. In addition, npmvCBGPS has a formal mechanism to guarantee that covariates will be balanced. This can explain why our estimator exhibits greater accuracy and precision compared to mvGPS. It is worth noting that we used the first-order moments of both the exposures and the covariates to assess covariate balance in this paper. Higher moments may be helpful when there are strong nonlinear correlations between exposures and covariates. However, the choices of moment orders should be made carefully, because using too many moments may lead to unstable weights [33, 39].

A valid inference is important for mixture exposure analysis. Here, we used a robust sandwich-type variance estimator as suggested by Joffe et al. [40]. The corresponding 95% CI coverage was close to the nominal value except when the sample size was 1000. We viewed this as a trade-off between bias and variance because npmvCBGPS focused on removing bias (the correlations between exposures and covariates are 0 after weighting) when estimating balancing weights [41]. How to account for bias and variance simultaneously, as done by Athey et al. when estimating balancing weights, is an area of future research [42].

The PFASs are endocrine-disrupting chemicals that can result in changes in metabolic outcomes such as BMI [22]. Several epidemiological studies have been instrumental in determining the potential health effects of exposure to PFASs on BMI [43–47]. However, the current evidence was inconclusive [37], possibly due to insufficient confounding control or the fact that the majority of these studies have focused on individual chemicals, ignoring coexposures. To this end, we applied npmvCBGPS to estimate the joint causal effects of exposure to PFASs on BMI and found an overall inverse trend of the PFAS mixtures with BMI. The results derived in this study were based on the publicly available datasets. Future large-scale prospective cohort studies are needed to consolidate the validity of these associations.

## Conclusions

In summary, this study proposed a robust statistical method, called npmvCBGPS, to estimate causal effects of multiple exposure mixtures on health outcomes for driving public health interventions and policy changes. A simulation study showed that npmvCBGPS outperformed the existing multivariate GPS (mvGPS) method in terms of accuracy, precision, and statistical testing in all scenarios. An appealing advantage of npmvCBGPS over the existing mvGPS method is robustness to model misspecification. This robustness enhances its applicability across various domains, with a particular emphasis on environmental epidemiology.

## Supplementary Information

Below is the link to the electronic supplementary material.

Supplementary Material 1

Supplementary Material 2

## Data Availability

No datasets were generated or analysed during the current study.
