# Supplementary material for: Estimating the causal effects of exposure mixtures: a generalized propensity score method
Source: BMC Med Res Methodol. 2025 Sep 29;25:221. doi: 10.1186/s12874-025-02673-4 (PMC12482879; doi:10.1186/s12874-025-02673-4)
Supplement: Supplementary file 2 — Supplementary Material 2 [file 12874_2025_2673_MOESM2_ESM.docx]

**Supporting Information**

Estimating the causal effects of exposure mixtures: A generalized propensity score method

*Qian Gao*^a^*, Ting Li*^a^*, Guiming Zhu*^a^*, Juping Wang*^a^*,* *Kexin Qiu*^c^*, Liangpo Liu*^b^*, Xiujuan Yang*^b^*, Tong Wang*^a*^

^a^ Department of Health Statistics, School of Public Health, MOE Key Laboratory of Coal Environmental Pathogenicity and Prevention, Shanxi Medical University, Taiyuan, China, 030001.

^b^ Department of Public Health Laboratory Sciences, School of Public Health, MOE Key Laboratory of Coal Environmental Pathogenicity and Prevention, Shanxi Medical University, Taiyuan, China, 030001.

^c^ Department of Statistics, The Chinese University of Hong Kong, Shatin, Hong Kong SAR, 999077.

**Correspondence to:* Tong Wang, Department of Health Statistics, School of Public Health, MOE Key Laboratory of Coal Environmental Pathogenicity and Prevention, Shanxi Medical University, No.56 Xinjian South Road, 030001, Taiyuan, China; Tel: +86-351-4135397; Fax: +86-351-4135998, Email: [tongwang@sxmu.edu.cn](mailto:tongwang@sxmu.edu.cn).

[Table S1 2](#_Toc204015073)

[Table S2 2](#_Toc204015073)

[Figure S1 3](#_Toc204015074)

[Figure S2 4](#_Toc204015075)

[Figure S3 5](#_Toc204015076)

[Figure S4 6](#_Toc204015077)

[Figure S5 7](#_Toc204015078)

[Figure S6 8](#_Toc204015079)

[Figure S7 9](#_Toc204015080)

[Figure S8 10](#_Toc204015081)

[Figure S9 11](#_Toc204015082)

[Figure S10 12](#_Toc204015083)

[Figure S11 13](#_Toc204015084)

[Figure S12 14](#_Toc204015085)

[Figure S13 14](#_Toc204015086)

# Table S1 Simulation Scenarios. Exposures *T* were generated as $\boldsymbol{N}_{\mathbf{3}}\left( m\left( \boldsymbol{X} \right)\mathbf{,}\boldsymbol{M} \right)\boldsymbol{,M=}\left( \begin{matrix} 1 & Trho=0.2 & Trho=0.2 \\ Trho=0.2 & 1 & Trho=0.2 \\ Trho=0.2 & Trho=0.2 & 1 \end{matrix} \right)$, and outcome *Y* was generated as $\boldsymbol{N}\left( \boldsymbol{\eta T+g}\left( \boldsymbol{X} \right)\boldsymbol{,1} \right)$.

| **Scenarios** | | **Covariates (*X*)** | | ***n*** | **Settings** | | $\boldsymbol{m}\left( \boldsymbol{X} \right)$ **(exposures)** | $\boldsymbol{g}\left( \boldsymbol{X} \right)$ **(outcome)** |
| --- | --- | --- | --- | --- | --- | --- | --- | --- |
| 1 | $\left( X_{1},X_{3},X_{4},X_{5},X_{6} \right)$  $\sim N_{5}\left( 0,\sum\right)$  $\Sigma_{ij}=1$ ($i=j$)  $\Sigma_{ij}=0.2$($i\neq j$)  $X_{2}\sim Bernoulli (p_{2}=0.5)$ | | 200,  500,  1000 | | | E1Y1 | $\boldsymbol{X}\boldsymbol{\alpha}_{1}$, $\boldsymbol{\alpha}_{1}=\left( \begin{aligned} 1,1,0,1,1,0 \\ 1,0,1,1,0,1 \\ 1,0,0,0,0,0 \end{aligned} \right)$ | ${0.6T}_{1}-0.5T_{2}+{0.8T}_{3}+\mathbf{X}\boldsymbol{\beta}_{\boldsymbol{1}}$  $\boldsymbol{\beta}_{\boldsymbol{1}}\boldsymbol{=}\left( 1,1,1,0,0,0 \right)$ |
|  |  | |  | | | E1Y2 |  | ${0.6T}_{1}-0.5T_{2}+{0.8T}_{3}+0.2T_{1}T_{3}+\boldsymbol{X}\boldsymbol{\beta}_{\boldsymbol{1}}$  $\boldsymbol{\beta}_{\boldsymbol{1}}\boldsymbol{=}\left( 1,1,1,0,0,0 \right)$ |
| 2 |  | |  | | | E1Y3 | $\boldsymbol{X}\boldsymbol{\alpha}_{1}$, $\boldsymbol{\alpha}_{1}=\left( \begin{aligned} 1,1,0,1,1,0 \\ 1,0,1,1,0,1 \\ 1,0,0,0,0,0 \end{aligned} \right)$ | ${0.6T}_{1}-0.5T_{2}+{0.8T}_{3}+\boldsymbol{X}\boldsymbol{\beta}_{1}+(\boldsymbol{X}*\boldsymbol{X})\boldsymbol{\beta}_{2}$  $\boldsymbol{\beta}_{\mathbf{1}}=\left( 1,1,1,0,0,0 \right)$, $\boldsymbol{\beta}_{\mathbf{2}}=\left( 1,0,0,0,0,0 \right)$ |
|  |  | |  | | | E1Y4 |  | ${0.6T}_{1}-0.5T_{2}+{0.8T}_{3}+0.2T_{1}T_{3}+\boldsymbol{X}\boldsymbol{\beta}_{\mathbf{1}}+(\boldsymbol{X}*\boldsymbol{X})\boldsymbol{\beta}_{\mathbf{2}}$  $\boldsymbol{\beta}_{\mathbf{1}}=\left( 1,1,1,0,0,0 \right)$, $\boldsymbol{\beta}_{\mathbf{2}}=\left( 1,0,0,0,0,0 \right)$ |
|  |  | |  | | | E2Y1 | $\boldsymbol{X}\boldsymbol{\alpha}_{1}+(\boldsymbol{X}*\boldsymbol{X})\boldsymbol{\alpha}_{\mathbf{2}}$  $\boldsymbol{\alpha}_{1}=\left( \begin{aligned} 1,1,0,1,1,0 \\ 1,0,1,1,0,1 \\ 1,0,0,0,0,0 \end{aligned} \right)$;  $\boldsymbol{\alpha}_{2}=\left( \begin{aligned} \begin{aligned} 1,0,0,0,0,0 \\ 1,0,0,0,0,0 \end{aligned} \\ 1,0,0,0,0,0 \end{aligned} \right)$ | ${0.6T}_{1}-0.5T_{2}+{0.8T}_{3}+\mathbf{X}\boldsymbol{\beta}_{\boldsymbol{1}}$  $\boldsymbol{\beta}_{\boldsymbol{1}}\boldsymbol{=}\left( 1,1,1,0,0,0 \right)$ |
|  |  | |  | | | E2Y2 |  | ${0.6T}_{1}-0.5T_{2}+{0.8T}_{3}+0.2T_{1}T_{3}+\boldsymbol{X}\boldsymbol{\beta}_{\boldsymbol{1}}$  $\boldsymbol{\beta}_{\boldsymbol{1}}\boldsymbol{=}\left( 1,1,1,0,0,0 \right)$ |
|  |  | |  | | | E2Y3 |  | ${0.6T}_{1}-0.5T_{2}+{0.8T}_{3}+\boldsymbol{X}\boldsymbol{\beta}_{1}+(\boldsymbol{X}*\boldsymbol{X})\boldsymbol{\beta}_{2}$  $\boldsymbol{\beta}_{\mathbf{1}}=\left( 1,1,1,0,0,0 \right)$, $\boldsymbol{\beta}_{\mathbf{2}}=\left( 1,0,0,0,0,0 \right)$ |
|  |  | |  | | | E2Y4 |  | ${0.6T}_{1}-0.5T_{2}+{0.8T}_{3}+0.2T_{1}T_{3}+\boldsymbol{X}\boldsymbol{\beta}_{\mathbf{1}}+(\boldsymbol{X}*\boldsymbol{X})\boldsymbol{\beta}_{\mathbf{2}}$  $\boldsymbol{\beta}_{\mathbf{1}}=\left( 1,1,1,0,0,0 \right)$, $\boldsymbol{\beta}_{\mathbf{2}}=\left( 1,0,0,0,0,0 \right)$ |

# Table S2 Results of linear model associating PFASs and BMI by npmvCBGPS

|  | $\beta$ (95% CI) | t | *P* |
| --- | --- | --- | --- |
| Intercept | 30.334 (29.542, 31.125) | 75.1325216 | <0.001 |
| lnPFHxS | -0.351 (-0.564, -0.139) | -3.243453027 | 0.001 |
| lnPFNA | 0.335 (0.087, 0.583) | 2.643542951 | 0.008 |
| lnPFOA | -0.396 (-0.893, 0.102) | -1.559154492 | 0.119 |
| lnPFOS | -0.319 (-0.567, -0.070) | -2.515197506 | 0.012 |

Note: CI: confidence interval; PFOA: perfluorooctanoic acid; PFOS: perfluorooctane sulfonic acid; PFHxS: perfluorohexane sulfonic acid; PFNA: perfluorononanoic acid;


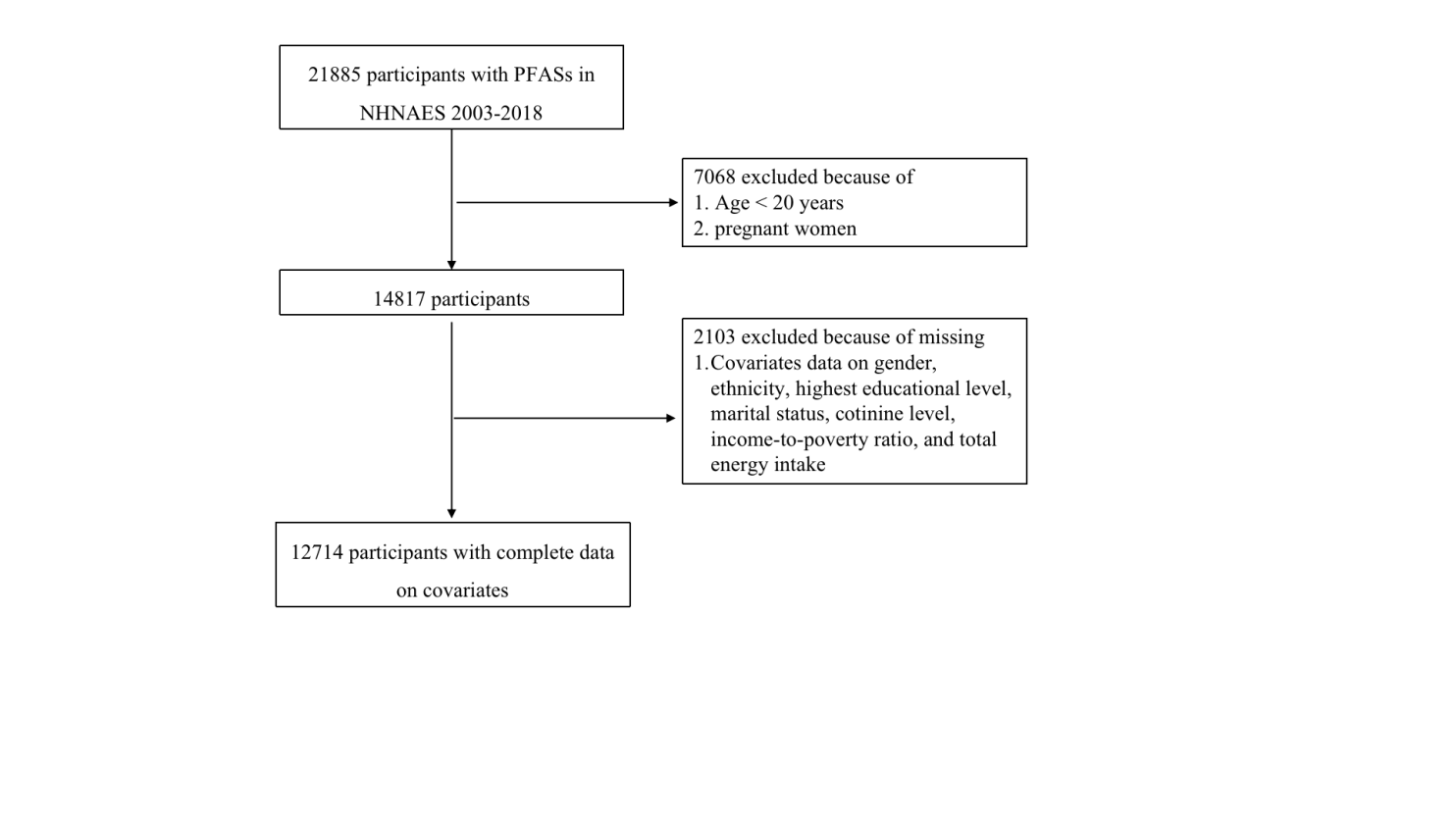


# Figure S1 Participant flowchart, NHANES 2003–2018


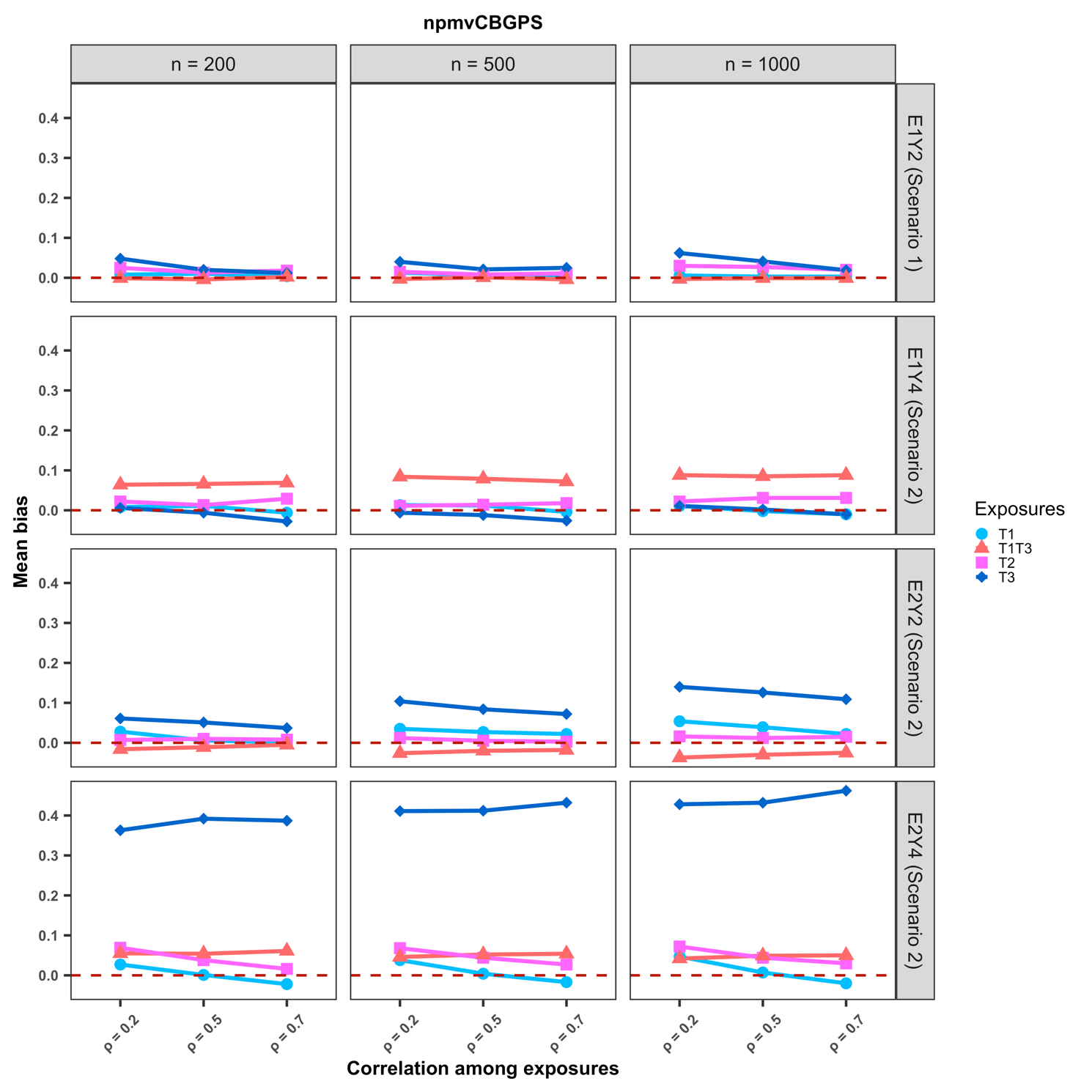


# Figure S2 Mean biases of causal parameter estimates at varying exposure correlations for the npmvCBGPS method in cases where there was an interaction among exposures


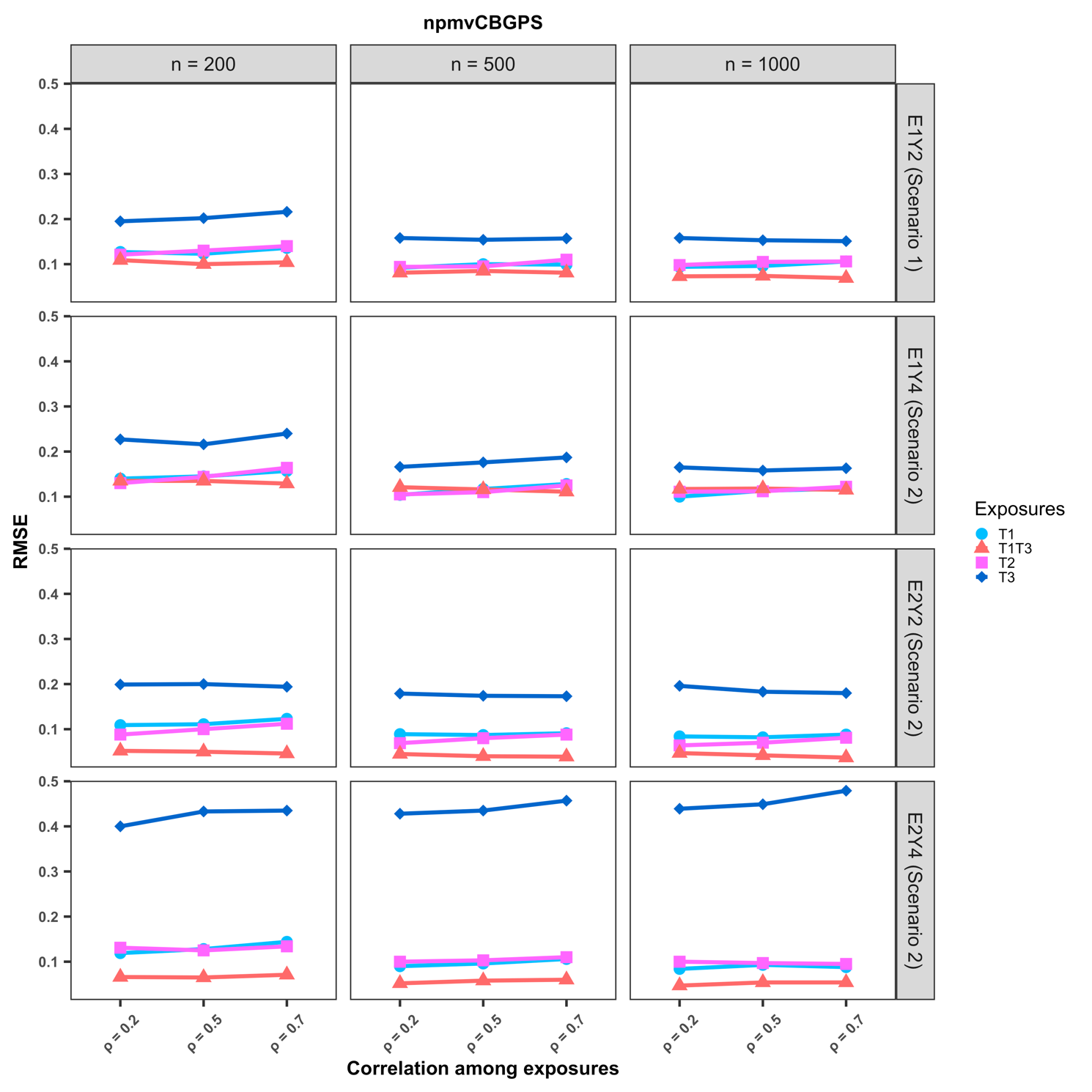


# Figure S3 RMSE of causal parameter estimates at varying exposure correlations for the npmvCBGPS method in cases where there was an interaction among exposures


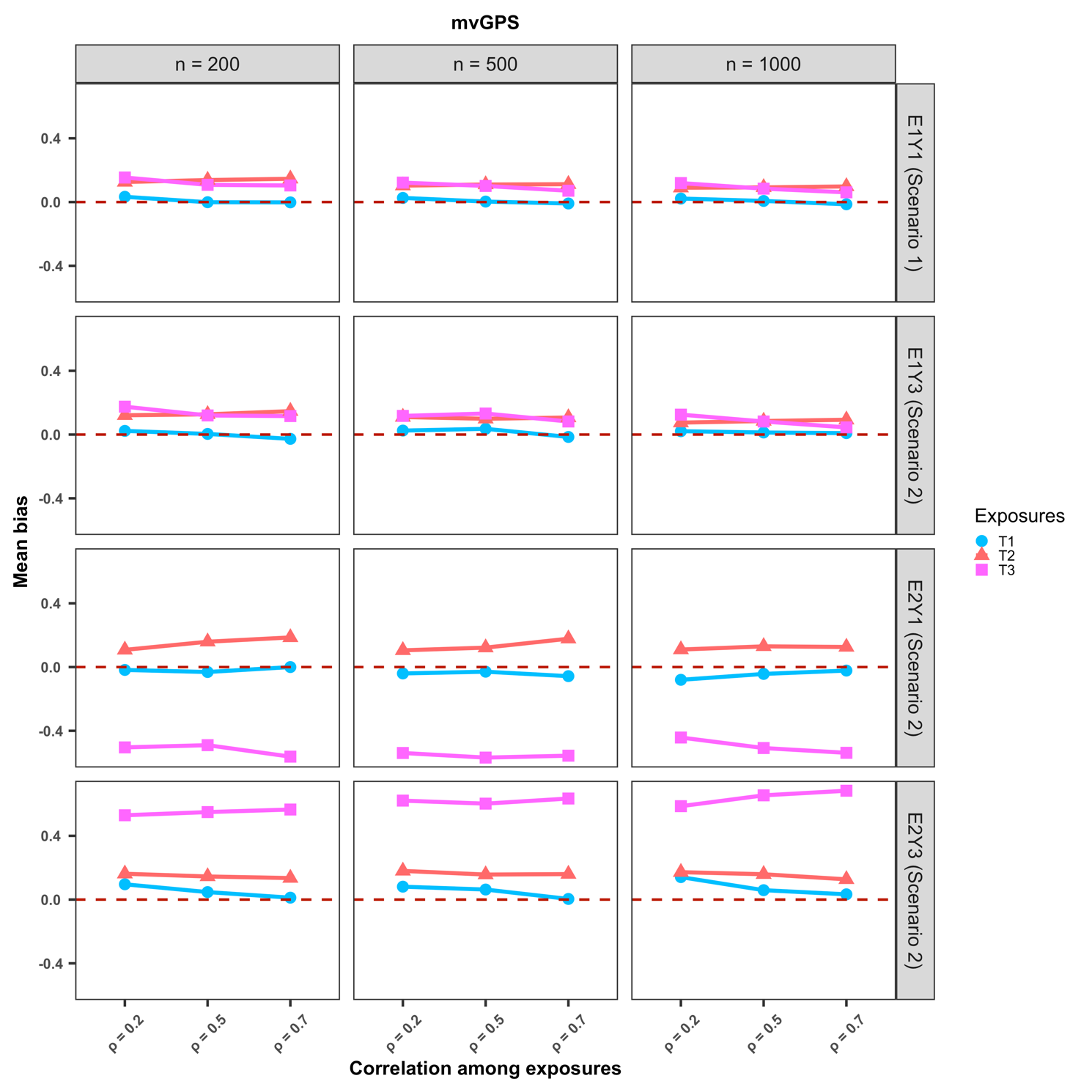


# Figure S4 Mean biases of causal parameter estimates at varying exposure correlations for the mvGPS method in cases where there was no interaction among exposures


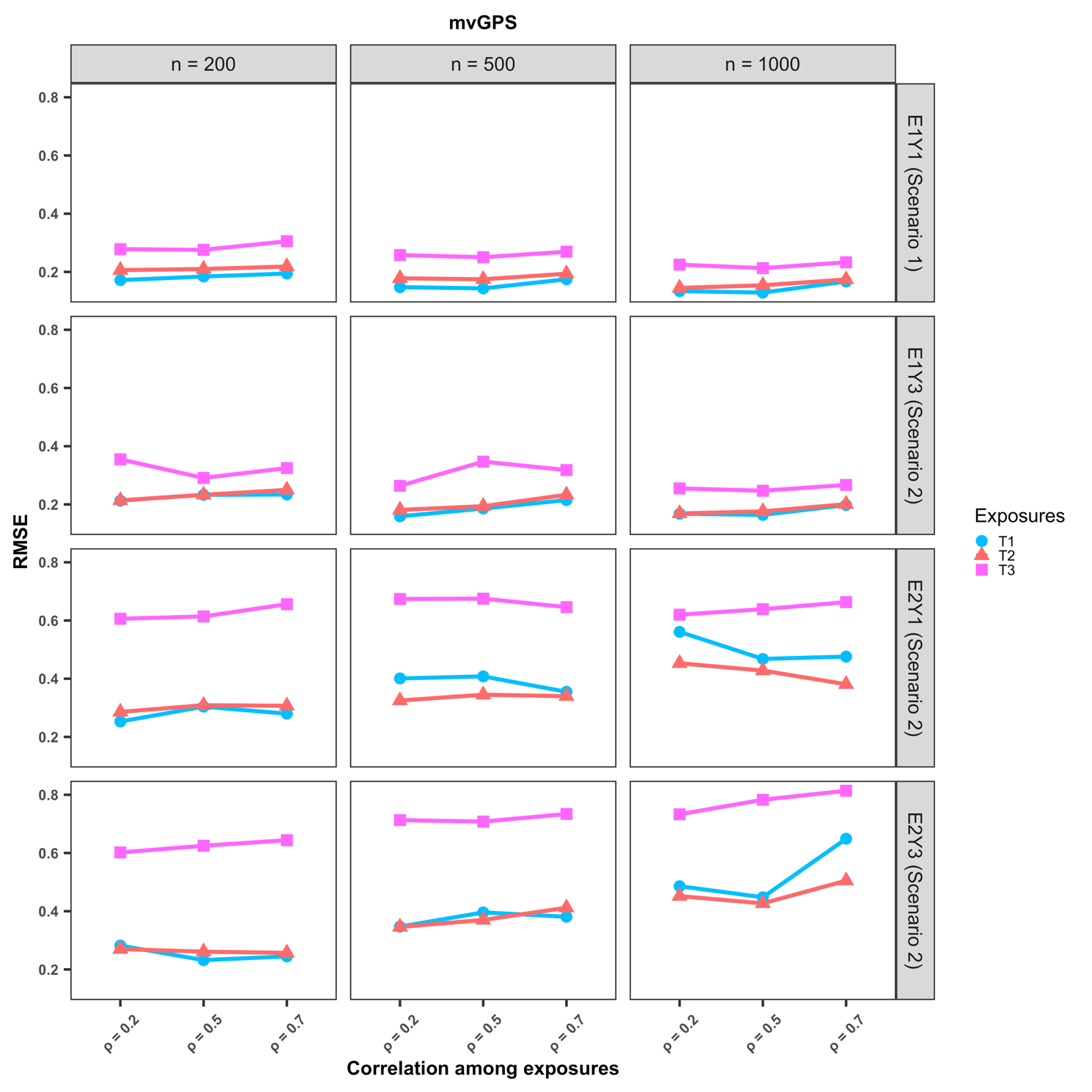


# Figure S5 RMSE of causal parameter estimates at varying exposure correlations for the mvGPS method in cases where there was no interaction among exposures


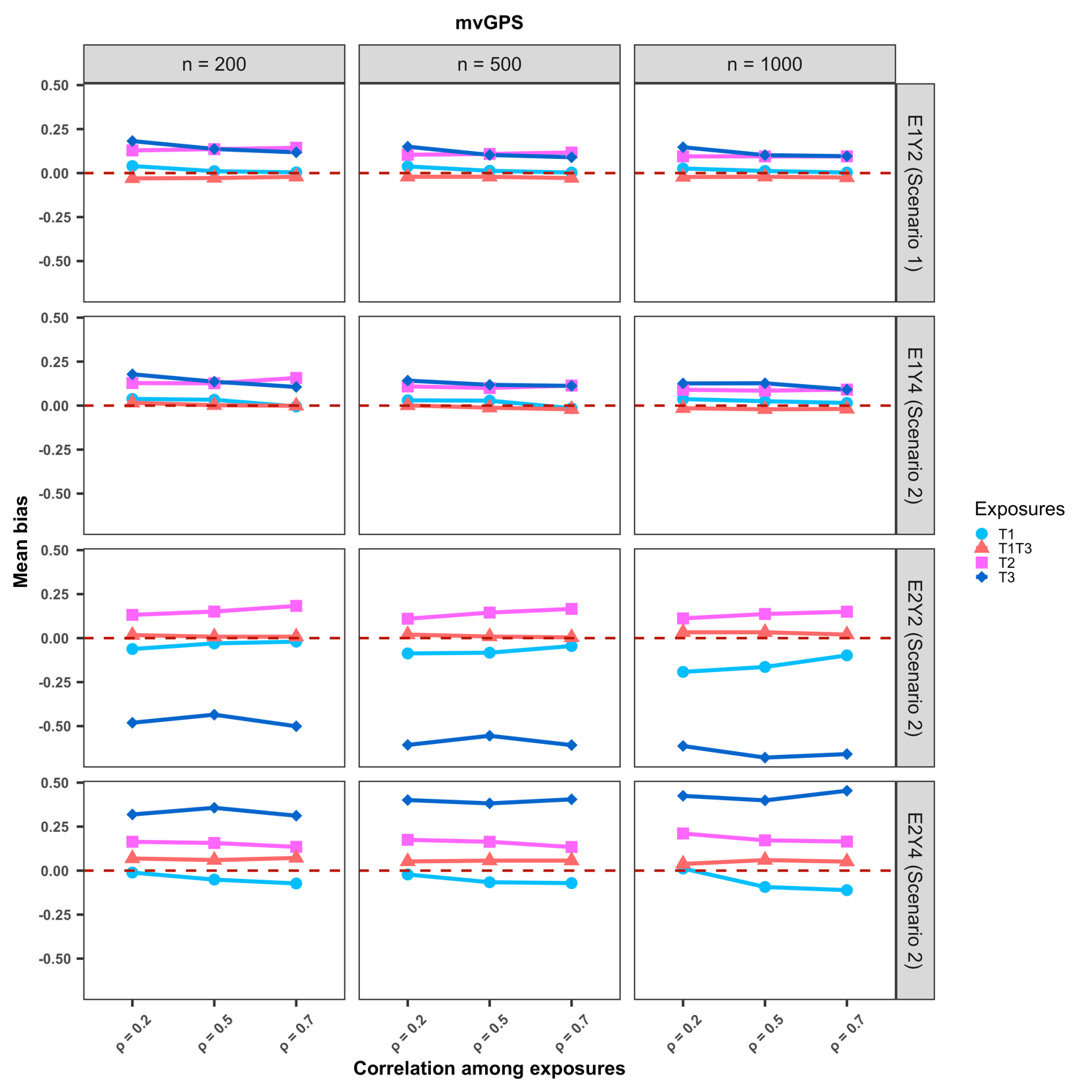


# Figure S6 Mean biases of causal parameter estimates at varying exposure correlations for the mvGPS method in cases where there was an interaction among exposures


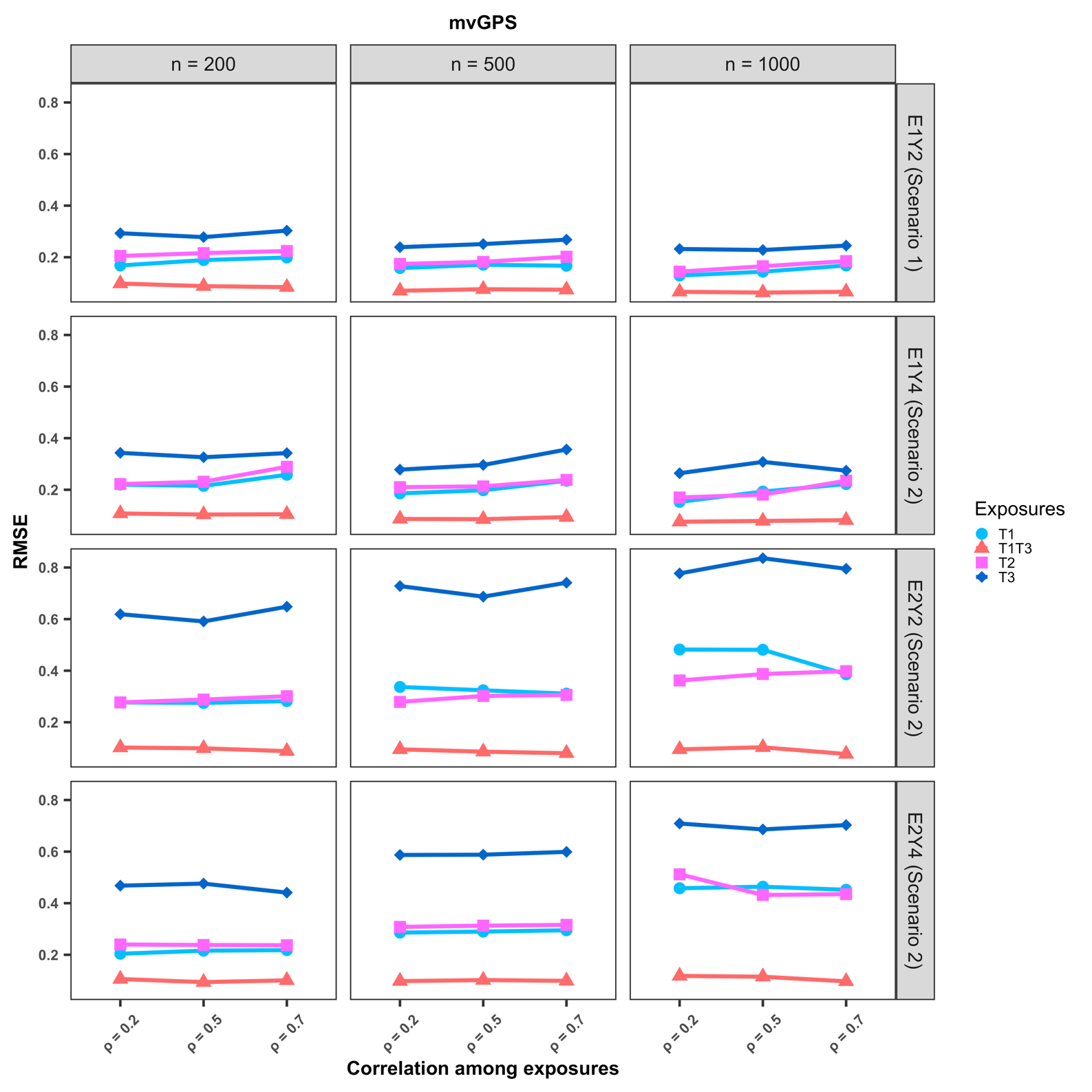


# Figure S7 RMSE of causal parameter estimates at varying exposure correlations for the mvGPS method in cases where there was interaction among exposures


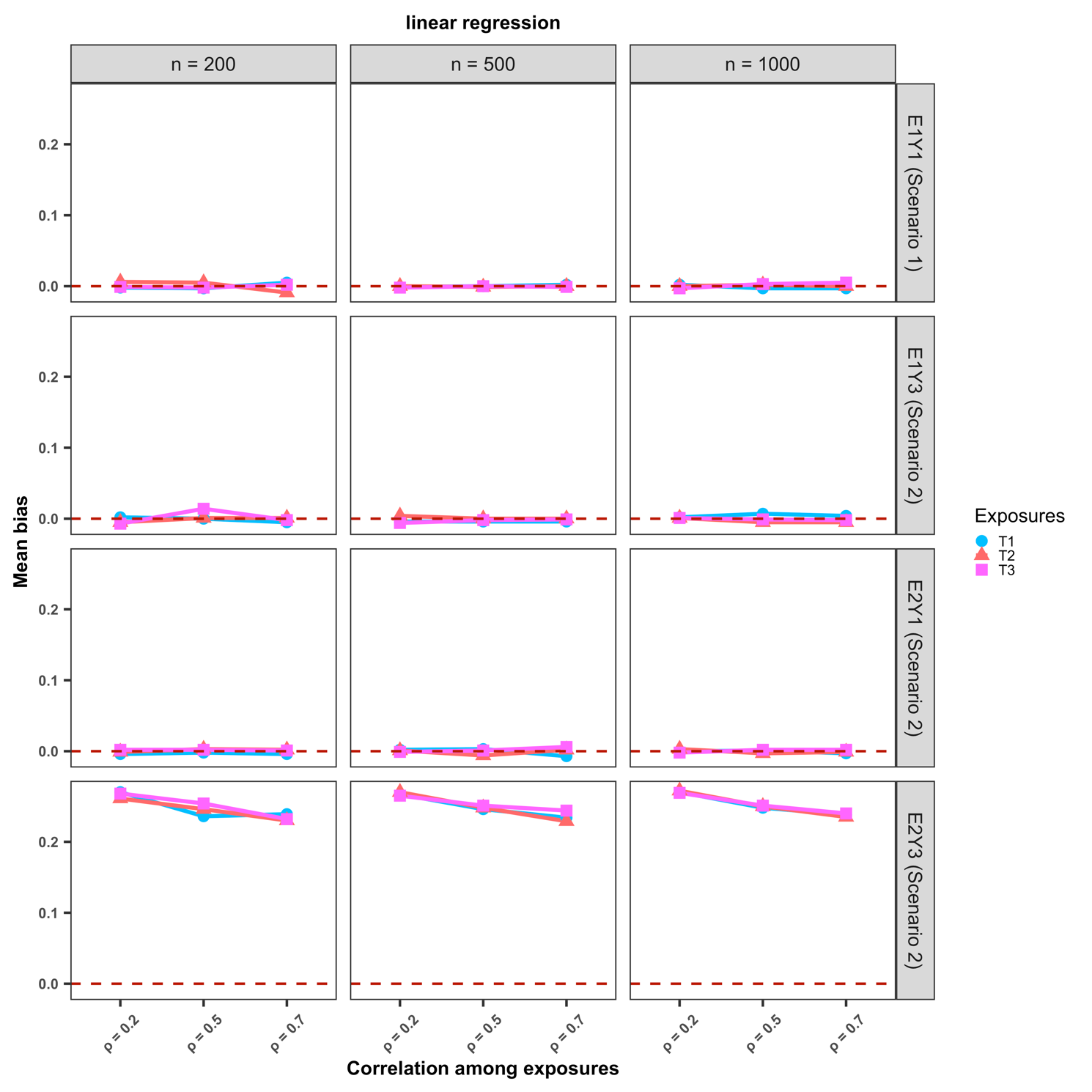


# Figure S8 Mean biases of causal parameter estimates at varying exposure correlations for the linear regression model in cases where there was no interaction among exposures


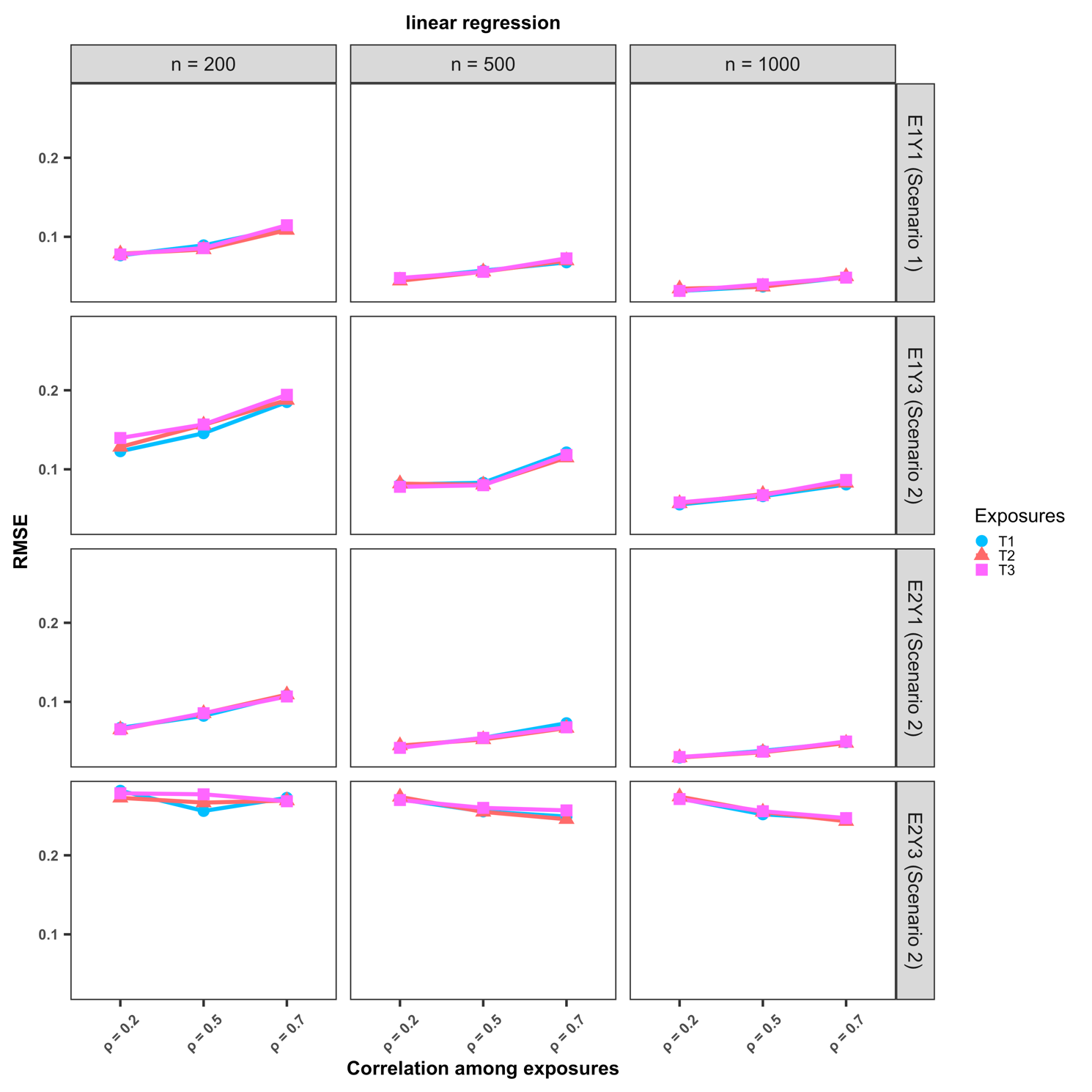


# Figure S9 RMSE of causal parameter estimates at varying exposure correlations for the linear regression model in cases where there was no interaction among exposures


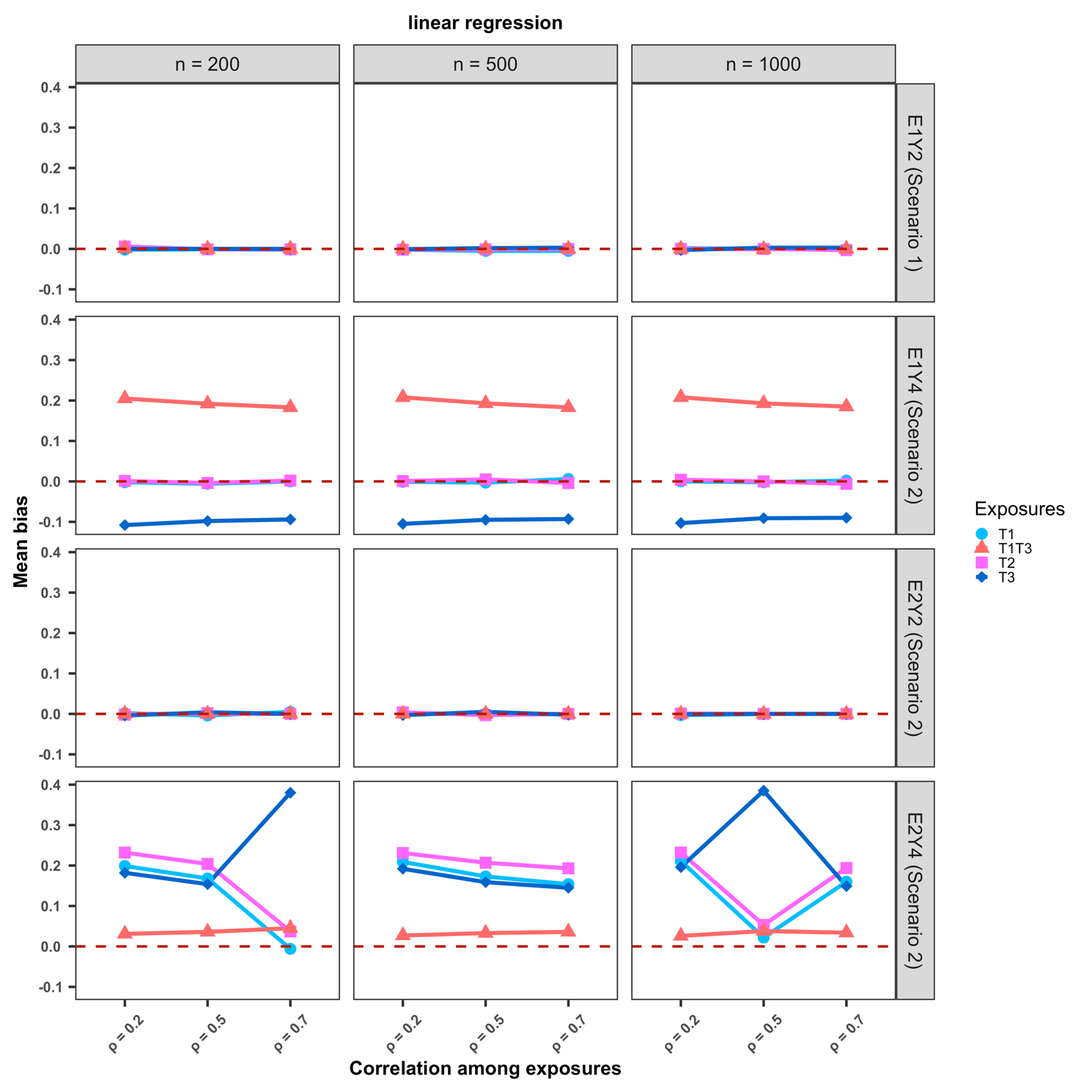


# Figure S10 Mean biases of causal parameter estimates at varying exposure correlations for the linear regression model in cases where there was an interaction among exposures


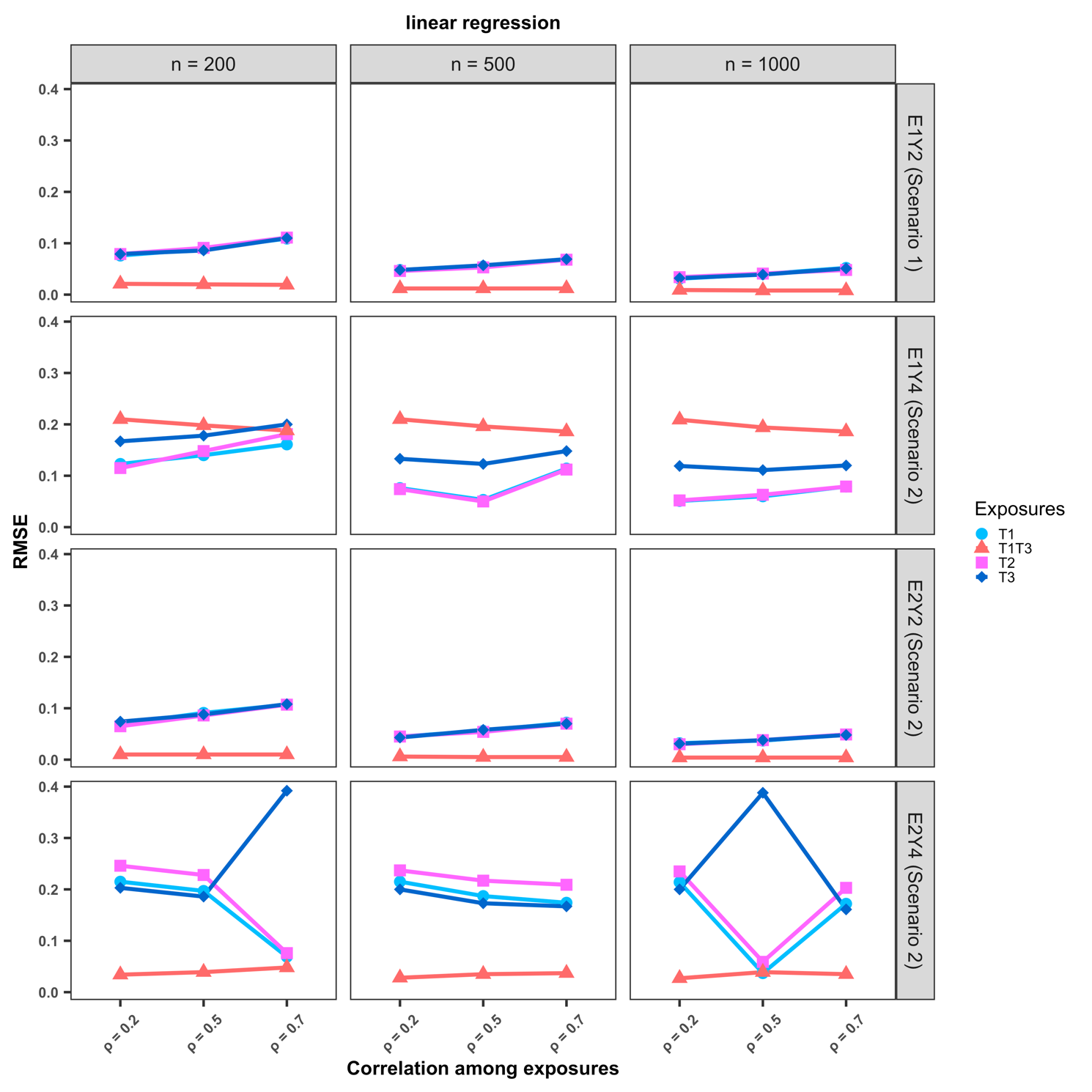


# Figure S11 RMSE of causal parameter estimates at varying exposure correlations for the linear regression model in cases where there was an interaction among exposures


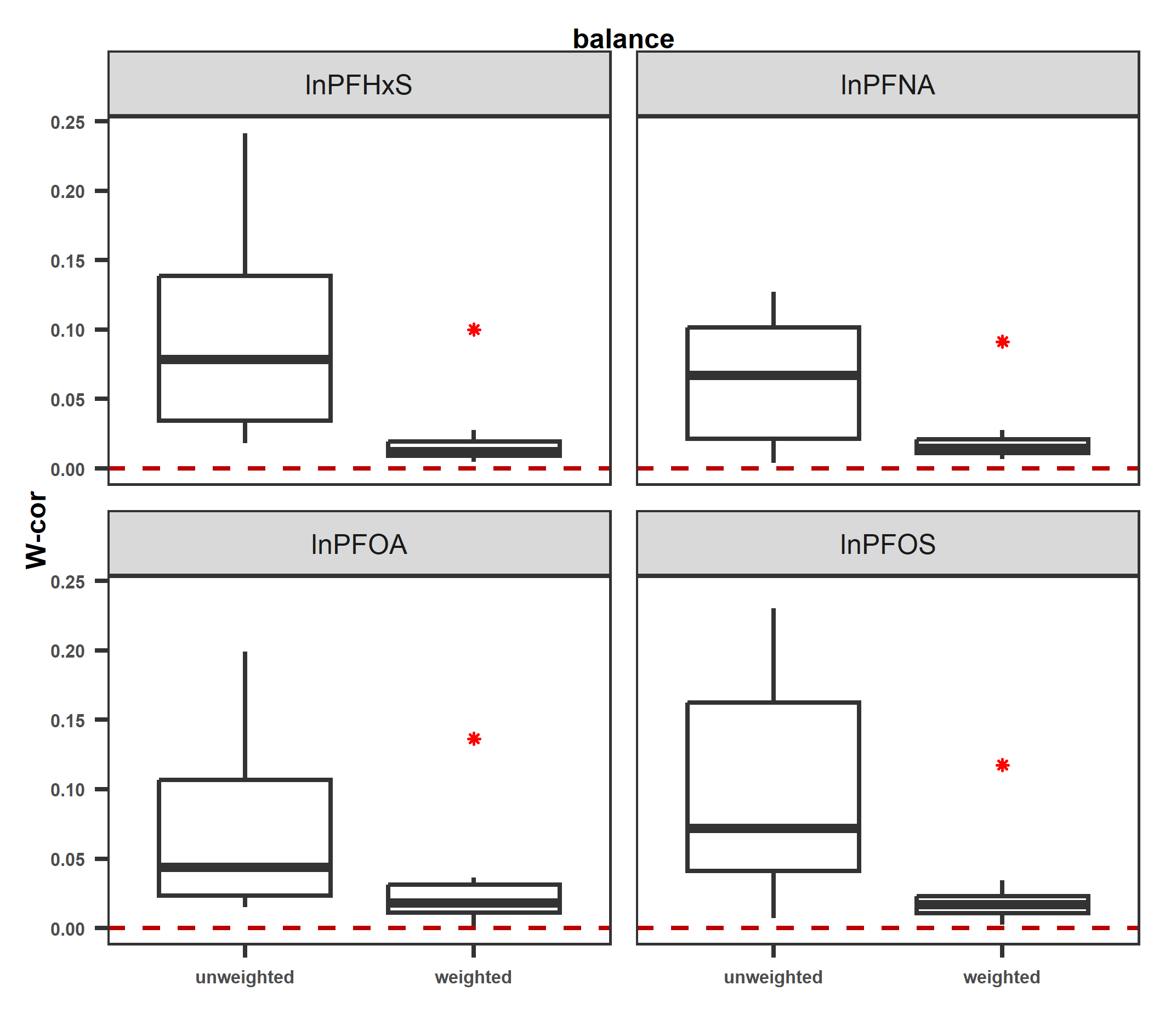


# **Figure S12** Covariate balance performance of npmvCBGPS for each component of Perfluoroalkyl and Polyfluoroalkyl Substances (PFASs)


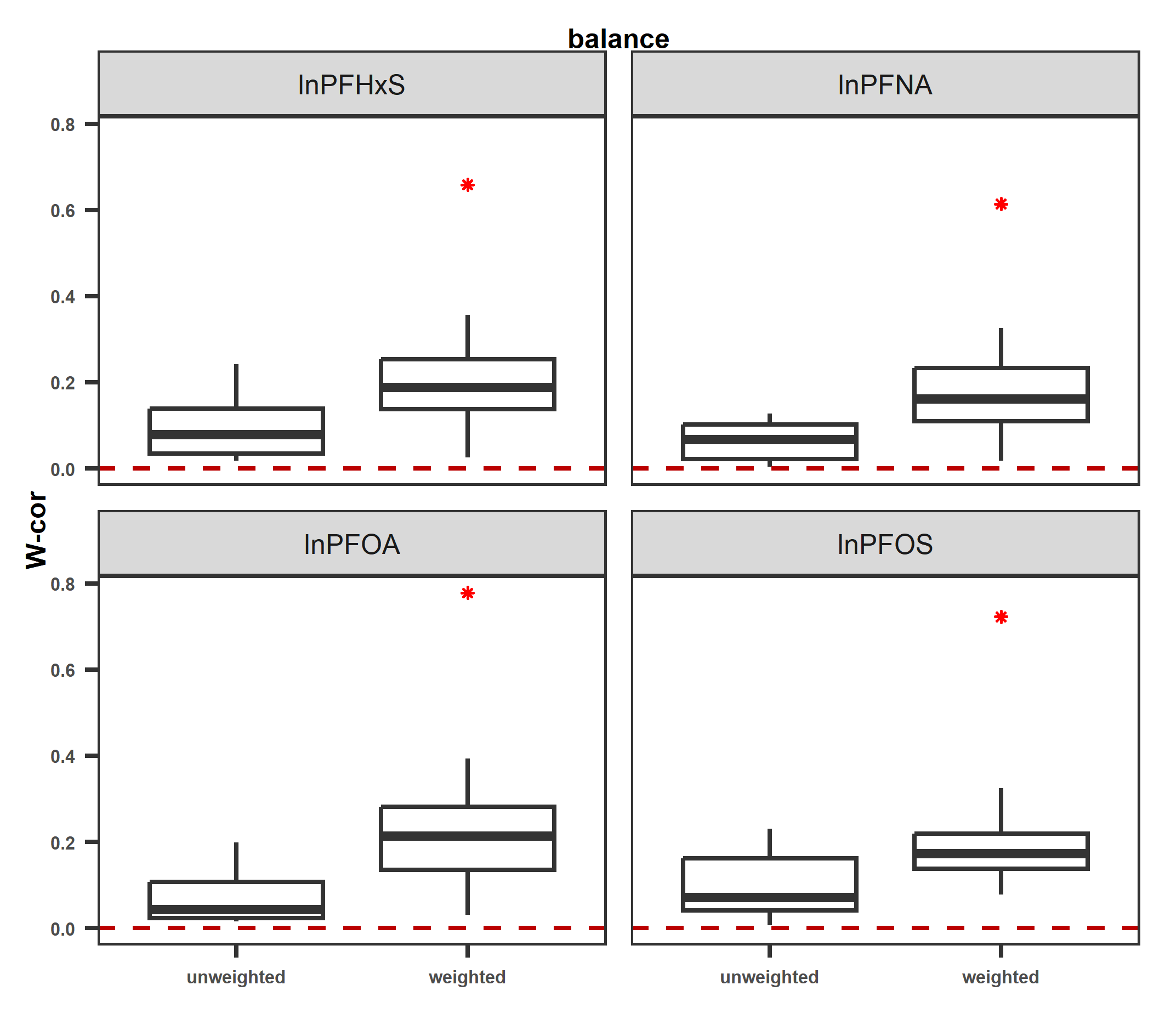


# **Figure S13** Covariate balance performance of mvGPS for each component of Perfluoroalkyl and Polyfluoroalkyl Substances (PFASs)
